# Activated CaSO_4_-Induced Vacuolation as a Quantitative Platform for Phagocytosis-Driven Drug Screening

**DOI:** 10.3390/molecules31214022

**Published:** 2026-11-08

**Authors:** Vishakha Goswami, Abul Faiz, Gaurav Dutt, Saima Bashir, Arushi Gupta, Snehasish Das, Apurba Gouri, Alpana Joshi, Subrata K. Das

**Affiliations:** 1 School of Biomedical Engineering and Health Sciences, Shobhit Institute of Engineering and Technology, NH 58, Meerut 250110, India; vishakha.goswami@shobhituniversity.ac.in (V.G.); abul.faiz@shobhituniversity.ac.in (A.F.); gaurav.dutt@shobhituniversity.ac.in (G.D.); saimashaikhh2000@gmail.com (S.B.); arushi18272944@gmail.com (A.G.); snehasish98320@gmail.com (S.D.); 2 Department of Chemistry, Jeonbuk National University, Jeonju 54896, Republic of Korea; apurbagouri@gmail.com

**Keywords:** activated calcium sulfate (ACS), gypsum, Godanti Bhasma (GB), phagosome, cytoplasmic vacuoles, phagocytosis, Bafilomycin A1

## Abstract

Cytoplasmic vacuolization is associated with phagocytosis, lysosomal acidification, and autophagy, yet robust in vitro models for its quantification and pharmacological screening remain limited. Here, we demonstrate that thermally activated calcium sulfate (ACS) induces extensive vacuolization across mammalian cell lines, including HeLa, RAW 264.7, 3T3-L1, and SH-SY5Y, establishing a versatile platform for studying vacuole biogenesis. To improve reproducibility, ACS particle heterogeneity was addressed by sedimentation-based fractionation, with the 5 min fraction producing homogeneous suspensions and stable, consistent vacuole formation. Vacuolization was quantified using Neutral Red (NR) uptake, and dose- and time-dependent analyses demonstrated a direct relationship between ACS concentration and vacuole induction. The assay was validated using bafilomycin A1 (BFA1), a selective V-ATPase inhibitor, which demonstrated concentration- and time-dependent inhibition of vacuole formation and acidification. Screening of ten commercially available drugs revealed distinct responses, including early cytotoxicity, strong vacuole inhibition, partial suppression, or negligible effects. The ability to distinguish vacuole inhibition from cytotoxic responses highlights the utility of ACS-induced vacuolization as a sensitive and scalable in vitro platform for mechanistic studies and functional screening of modulators of lysosomal and phagocytic pathways relevant to infection, lysosomal disorders, and phagocytic dysfunction.

## 1. Introduction

Phagocytosis is essential for host defense, clearance of cellular debris, tissue homeostasis, and immune regulation. This process involves receptor-mediated particle recognition, actin-dependent engulfment, phagosome formation, and subsequent maturation through interactions with endosomal and lysosomal compartments [1,2,3,4,5,6,7]. Dysregulation of phagocytic processes contributes to diverse pathological conditions. Defective clearance via phagocytosis and efferocytosis is associated with chronic inflammation, autoimmune disease, atherosclerosis, neurodegeneration, and impaired tissue repair, whereas excessive or dysregulated particle engulfment can contribute to pathological tissue responses and immune-mediated disorders. In tumors, altered phagocytic pathways can influence immune surveillance, immune evasion, and tumor progression [8,9,10,11,12,13,14,15]. Therapeutic strategies may therefore require either enhancement or suppression of phagocytic activity depending on the disease context, highlighting the need for reliable quantitative assays for identifying and characterizing pharmacological modulators of particle uptake and intracellular processing. Screening of approved drugs has already proven useful for finding phagocytosis modulators, for example cardiac glycosides that potentiate CD47 blockade [16] and, in a recent preprint, histone deacetylase (HDAC) inhibitors that enhance phagocytosis by patient-derived tumor-associated macrophages [17]. Drug repurposing also offers shorter development timelines because safety data already exist [18,19].

Current in vitro phagocytosis assays commonly use fluorescently labelled bacteria, yeast, latex or polymeric beads, pH-sensitive particles, microscopy, flow cytometry, or high-content imaging [20,21,22,23,24,25,26,27]. These approaches provide valuable information on particle uptake, localization, and phagosome maturation but frequently require labelled targets or fluorescent probes, specialized reagents and instrumentation, and workflows involving particle preparation, labelling, washing, imaging, or flow-cytometric analysis [28,29]. Microscopy-based approaches can also become labor-intensive when large numbers of cells or compounds are evaluated, whereas automated high-content imaging can improve throughput but remains dependent on specialized imaging platforms and, in many cases, labelled targets [22,23,24,25,26,27]. These requirements increase assay complexity and cost and may limit the practicality of conventional approaches for large-scale pharmacological screening. Moreover, pharmacological agents can act at distinct stages of phagocytosis, including particle recognition, engulfment, phagosome formation, maturation, acidification, and intracellular degradation. Thus, improved quantitative assays are needed to facilitate the identification of compounds that modulate these processes. A simple and robust cellular readout of particle-induced intracellular responses could therefore complement conventional uptake-based assays and facilitate pharmacological screening.

An alternative approach is to quantify a cellular response generated following particle internalization rather than directly labelling the phagocytic target. Particle internalization and subsequent trafficking through endosomal and lysosomal compartments can induce intracellular vacuolization and remodeling of acidic compartments [30,31,32,33]. During maturation of these intracellular compartments, progressive acidification is mediated, in part, by vacuolar H^+^-ATPase (V-ATPase)-dependent proton transport [34,35,36]. Neutral red (NR), a weakly basic dye that preferentially accumulates in acidic intracellular compartments, can therefore provide an absorbance-based measure of intracellular acidification and associated vacuolar responses. This property enables particle-induced changes in acidic compartments to be quantified using a standard microplate format without requiring fluorescent labelling of the particle. Bafilomycin A1, an inhibitor of V-ATPase activity, therefore provides a pharmacological reference for assessing the contribution of V-ATPase-dependent acidification to the assay response [35,36,37,38,39].

Here, we developed biocompatible activated calcium sulfate (ACS) microparticles that are efficiently internalized by mammalian cells and induce robust intracellular vacuolization. We exploited this response to establish an NR-based microplate assay in which ACS-induced changes in intracellular vacuolization and acidification are quantified by absorbance, without requiring fluorescent particle labelling or specialized imaging as the primary readout. The ACS-NR system provides a simple, quantitative, scalable, and cost-effective approach compatible with standard microplate workflows. Pharmacological perturbation of NR accumulation can further be used to evaluate changes in intracellular acidification and vacuolar responses, enabling concentration- and time-dependent analyses and pharmacological validation. As an initial proof-of-concept, ten commercially available pharmaceutical formulations representing diverse pharmacological classes were selected to evaluate whether the assay can detect differential effects on the ACS-induced vacuolar response. The formulations included non-steroidal anti-inflammatory and analgesic agents, namely aspirin, celecoxib (a COX-2-selective inhibitor), and aceclofenac–paracetamol–serratiopeptidase (Zerodol-SP and BE-Fame-SP); the lipid-lowering agent atorvastatin; the corticosteroid prednisolone acetate; the antidiabetic combination glimepiride–metformin; the antibacterial combination ofloxacin–ornidazole; and the adrenergic and antimuscarinic agents oxymetazoline and phenylephrine–tropicamide. The primary objective of the present study was to establish the feasibility of the assay rather than to investigate the pharmacological mechanisms of the individual drugs. Collectively, the results support the ACS-NR platform as a complementary cellular assay for examining particle-induced intracellular responses and for screening compounds that modulate phagocytosis-associated cellular processes.

## 2. Results and Discussion

### 2.1. Characterization of Anhydrous ACS via FT-IR and XRD

**Figure 1:**
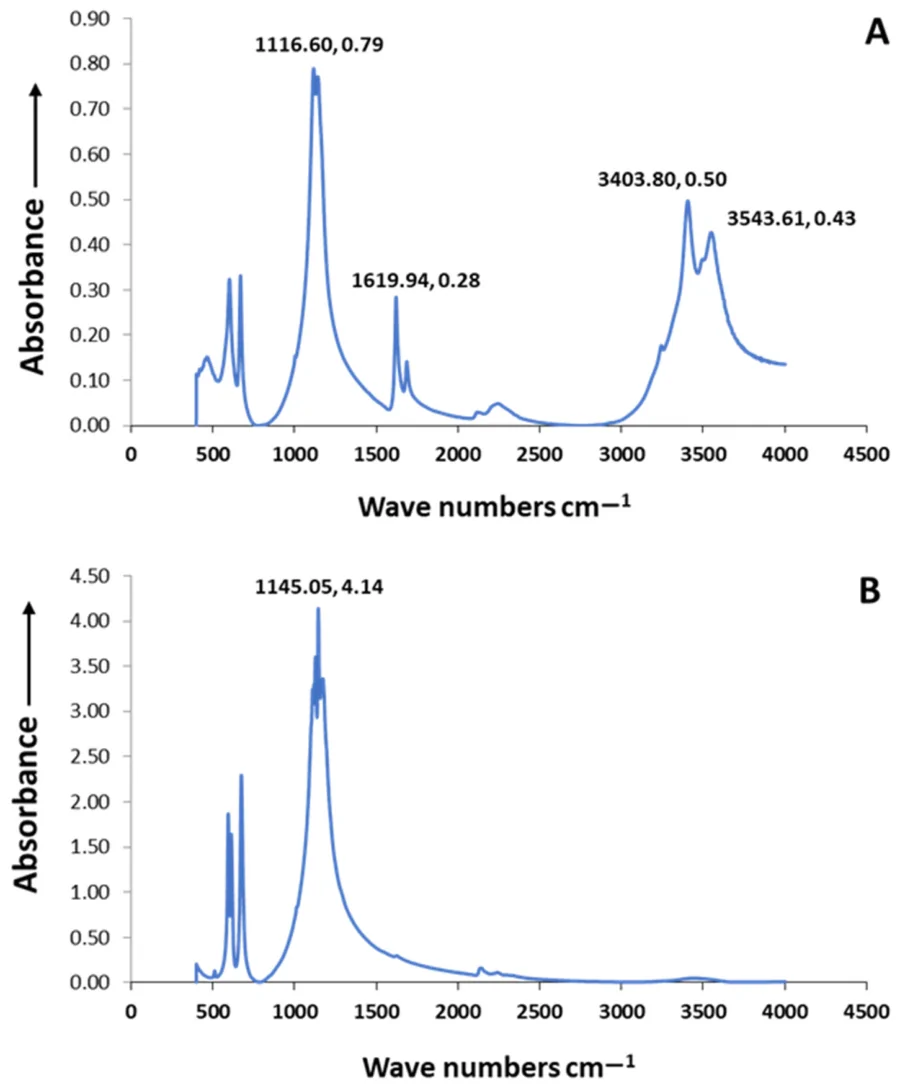
FTIR analysis of raw and heat-activated calcium sulfate. (**A**) FTIR spectrum of calcium sulfate dihydrate (CaSO_4_·2H_2_O) showing characteristic O–H stretching (3000–4000 cm^−1^) and bending (1600–1700 cm^−1^) vibrations corresponding to crystalline water, along with sulfate group (SO_4_^2−^) absorption bands around 1100–1150 cm^−1^. (**B**) FTIR spectrum of heat-activated calcium sulfate (CaSO_4_), demonstrating the disappearance of O–H vibrational bands, confirming the removal of crystalline water and transition to the anhydrous phase.

Thermal activation of calcium sulfate induced a clear dehydration-driven phase transition from gypsum (CaSO_4_·2H_2_O) to anhydrous activated calcium sulfate (CaSO_4_). FT-IR analysis provided direct chemical evidence of this transformation. Raw gypsum exhibited prominent O–H stretching and bending vibrations in the regions of 3000–4000 cm^−1^ and 1600–1700 cm^−1^, respectively, corresponding to hydrogen-bonded water molecules within the gypsum lattice (Figure 1A). In contrast, heat-activated calcium sulfate (ACS) showed complete disappearance of these O–H vibrational bands (Figure 1B), confirming the removal of crystalline water and formation of an anhydrous phase. This disappearance of O-H vibrational bands upon heating is a definitive indication of the removal of crystalline water molecules [40]. Characteristic sulfate (SO_4_^2−^) vibrational bands observed around 1100–1150 cm^−1^ were retained in both samples, with minor shifts in the activated material. The overall changes indicated the internal structural rearrangement and how it behaves physically and chemically [41].

**Figure 2:**
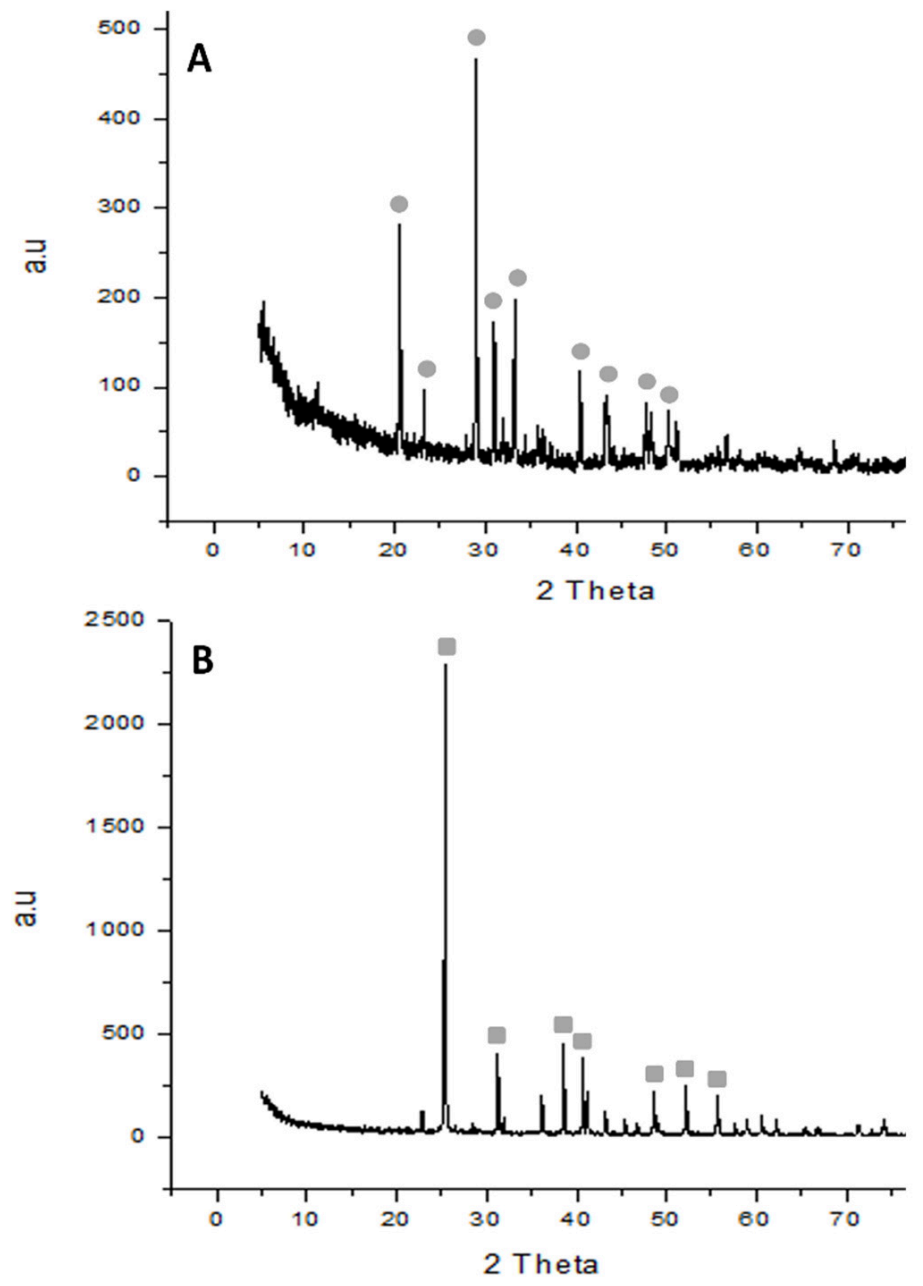
XRD patterns of hydrated and anhydrous calcium sulfate. (**A**) XRD profile of calcium sulfate dihydrate (CaSO_4_·2H_2_O) exhibiting diffraction peaks at 2θ values of 20.5°, 23.2°, 29°, 31°, 33.2°, 40.4°, 43.4°, 48°, and 50°, characteristic of the crystalline gypsum structure. (**B**) XRD profile of heat-activated anhydrous calcium sulfate (CaSO_4_), displaying distinct peaks at 2θ values of 25.2°, 31°, 36°, 38.2°, 40.9°, 48.4°, 52°, and 55.9°, confirming structural transformation to the anhydrite phase.

X-ray diffraction (XRD) serves as an indispensable tool for characterizing the crystalline phases of materials and monitoring phase transitions. The XRD spectrum was analyzed for both raw calcium sulfate dihydrate (CaSO_4_·2H_2_O) particles and heat-activated anhydrous calcium sulfate particles. For the dihydrated calcium sulfate, characteristic diffraction peaks were observed at 2θ values of 20.5°, 23.2°, 29°, 31°, 33.2°, 40.4°, 43.4°, 48°, and 50°, corresponding to the well-defined crystalline structure of gypsum (Figure 2A). These peaks arise due to the ordered arrangement of calcium, sulfate, and water molecules in the lattice. In contrast, the heat-activated anhydrous calcium sulfate displayed diffraction peaks at 2θ values of 25.2°, 31°, 36°, 38.2°, 40.9°, 48.4°, 52°, and 55.9°, indicating a structural transformation into an anhydrous phase (Figure 2B). The diffraction peaks observed for gypsum correspond to its monoclinic crystal system, with water molecules playing a pivotal role in maintaining lattice stability and symmetry [42,43,44]. Upon heating, the dehydration process results in significant structural rearrangements, giving rise to new peaks characteristic of anhydrite. The shift in peak positions and the emergence of new peaks in the anhydrous form signify a reduction in crystal symmetry, attributed to the collapse of the hydration framework [42,43,44,45].

### 2.2. Particle Heterogeneity/Homogeneity in Vacuole Biogenesis and Its Quantification

Particle heterogeneity is a critical determinant of cellular uptake dynamics and vacuolar responses, as variations in particle size, shape, and aggregation state directly influence phagocytic efficiency and intracellular processing [46]. One of the primary reasons for minimizing or systematically evaluating particle heterogeneity in ACS preparations is that excessive particle burden can obscure optical clarity, thereby hindering accurate visualization and assessment of intracellular vacuoles. In our previous work, we demonstrated that Godanti Bhasma (formulation of anhydrous CaSO_4_ with herbal ex-tract) induces massive vacuolation in mammalian cells, whereas gypsum fails to elicit comparable cellular responses [47]. In the present study, raw gypsum was coarsely powdered and thermally activated to generate activated calcium sulfate (ACS) micro-particles capable of inducing pronounced vacuolation in mammalian cells. However, the vacuolating activity of ACS was heterogeneous, likely reflecting variability in particle characteristics. Due to the mechanical nature of the preparation process, the resulting particle suspensions initially comprised a broad distribution of particle sizes.

**Figure 3:**
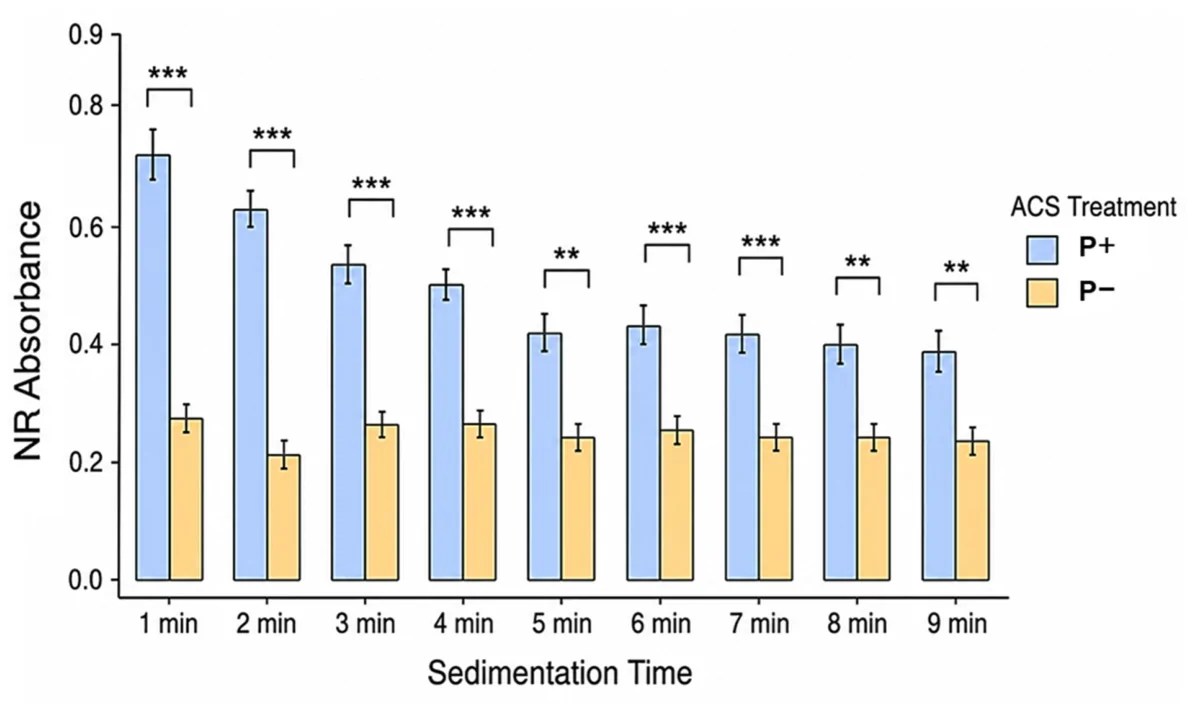
Correlation of particle homogeneity with reproducible vacuolation. HeLa cells exposed to early heterogeneous suspensions (1–3 min) exhibited pronounced and variable vacuolation, while later homogeneous suspensions (5–9 min) produced more consistent and moderate vacuolation patterns. P−, cells without ACS treatment; P+, cells treated with ACS. These results demonstrate the direct impact of particle heterogeneity on cellular uptake dynamics and vacuole formation (*n* = 3). Data are presented as mean ± SD (*n* = 3). Statistical significance was determined by two-way ANOVA with sedimentation time and ACS treatment as factors, followed by Tukey’s multiple-comparison test. ** *p* < 0.01, *** *p* < 0.001.

Microscopic examination was performed on ACS suspensions collected at each sedimentation time point, while neutral red (NR) staining was carried out on HeLa cells treated with ACS derived from the corresponding fractions. Early sedimentation fractions (1–3 min) contained highly heterogeneous particle populations, characterized by wide size variability and elevated particle numbers (Figure S1F–H). HeLa cells exposed to these early fractions exhibited extensive vacuolation, consistent with robust phagocytic uptake of particles spanning a broad size range (Figure S1A–C). From the 4 min time point onward, ACS preparations showed a progressive shift toward homogeneity, with particles displaying a more uniform size distribution. By the 5 min fraction, particle numbers stabilized, and suspensions remained consistent across sub-sequent time points (Figure S1I,J). Correspondingly, NR staining revealed that vacuolation in HeLa cells treated with ACS from the 5–9 min fractions were moderate, uniform, and highly reproducible (Figure 3). This sedimentation-based separation thus provided a temporal means of enriching for particles of different size classes. At early time points (1–3 min), incomplete sedimentation resulted in suspensions enriched in larger and more polydisperse particles. Such heterogeneity likely enhanced phagocytic activity, as cells encounter both larger particles that require extensive actin re-modelling and smaller particles that are rapidly internalized (Figure 3). The mixed particle load could trigger multiple uptake pathways simultaneously, producing pronounced vacuolation. In contrast, later fractions particularly the 5 min fraction—predominantly contain smaller, sedimentation-stable particles that produce a uniform uptake profile and consistent vacuolar responses. Based on these criteria, the 5 min sedimentation fraction was selected for all subsequent cellular experiments to ensure reproducibility and quantitative consistency.

**Figure 4:**
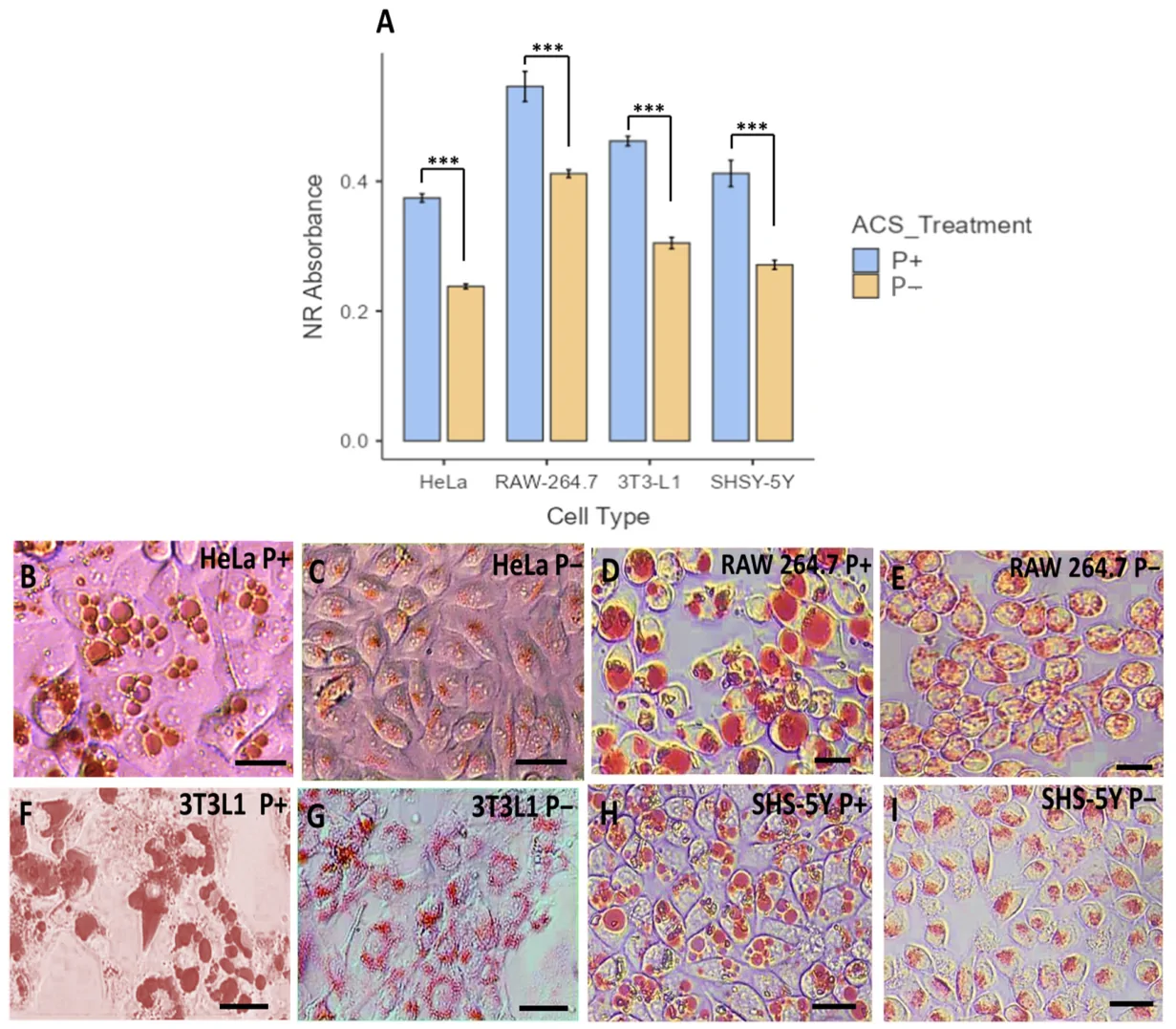
Quantitative and morphological assessment of ACS-induced cytoplasmic vacuolation across mammalian cell lines. (**A**) Quantitative analysis of intracellular vacuolation using the neutral red (NR) uptake assay. Cells treated with 5 min-sedimented activated calcium sulfate (ACS) (P+) stock exhibited a significant increase in NR accumulation compared to untreated controls (P−), indicating enhanced formation of acidic vacuolar compartments. Data are presented as mean ± SD (*n* = 4). Statistical significance was determined by two-way ANOVA with cell type and ACS treatment as fixed factors, followed by Tukey’s multiple-comparison test. *** *p* < 0.001, demonstrating reproducible induction of vacuolation upon ACS exposure. (**B**–**I**) Representative bright-field microscopy images illustrating NR-stained cells across multiple mammalian cell lines, including HeLa (**B**,**C**), RAW 264.7 macrophages (**D**,**E**), 3T3-L1 fibroblasts (**F**,**G**), and SH-SY5Y neuroblastoma cells (**H**,**I**). ACS-treated cells (PC; (**B**,**D**,**F**,**H**)) display extensive cytoplasmic vacuolation characterized by the presence of large, densely NR-positive vesicular structures, confirming their acidic and lysosome-like nature. These vacuoles are frequently associated with internalized particulate material, consistent with active phagocytic or endocytic processing. In contrast, untreated control cells (NC; (**C**,**E**,**G**,**I**)) exhibit minimal NR staining, limited to small, discrete lysosomal compartments, reflecting basal endo-lysosomal activity without significant vacuolar expansion. Scale bars: 20 µm (**B**,**C**), 15 µm (**D**–**G**) and 12 µm (**H**,**I**).

This method was established to quantify vacuolation across different cell lines (professional and non-professional phagocytic cells), HeLa, 3T3-L1, RAW-264.7, and SH-SY5Y cells (Figure 4B–I). Untreated cells contained only small lysosomal vacuoles, whereas 5th minute ACS stock-treated cells displayed abundant vacuoles that were stained with neutral red (NR), confirming their acidic nature [48]. Since lysosomal and vacuolar compartments maintain a lower pH than the cytoplasm, NR readily penetrates and ac-cumulates in these organelles [49]. Quantitative bar graph and statistically analysis of NR uptake further supported ACS-induced vacuolization (Figure 4A). In contrast to conventional phagocytic substrates such as latex beads or zymosan, ACS consistently induces extensive vacuolation characterized by large, optically clear, and stable phagosomes that are readily quantifiable [47,48]. These oversized vacuoles constitute a pronounced and reproducible cellular phenotype, permitting high-resolution observation of vacuole formation and acidification. Consequently, ACS provides a uniquely interpretable in vitro phagocytic model that is not attainable using commercially available particles. Furthermore, ACS exhibits strong compatibility with Neutral Red (NR) staining, wherein the formation of large vacuoles facilitates a simple and cost-effective assessment of vacuolar acidification. Consistent with this, NR robustly accumulated within ACS-induced vacuoles [47]. Hence, NR absorbance was interpreted as a quantitative measure of the acidic vacuolar response rather than as a direct measurement of vacuole number or particle uptake.

### 2.3. Concentration and Time-Dependent Cellular Effects of Activated Calcium Sulfate Particles

Quantification of ACS-induced vacuolation was performed using the neutral red (NR) uptake assay in HeLa cells treated with the 5 min ACS sedimentation fraction [49]. A clear dose-dependent response was observed, with vacuole formation increasing progressively with rising concentrations of ACS particles. Vacuole formation and acidification were assessed using Neutral Red staining, which preferentially labels acidic intracellular compartments.

**Figure 5:**
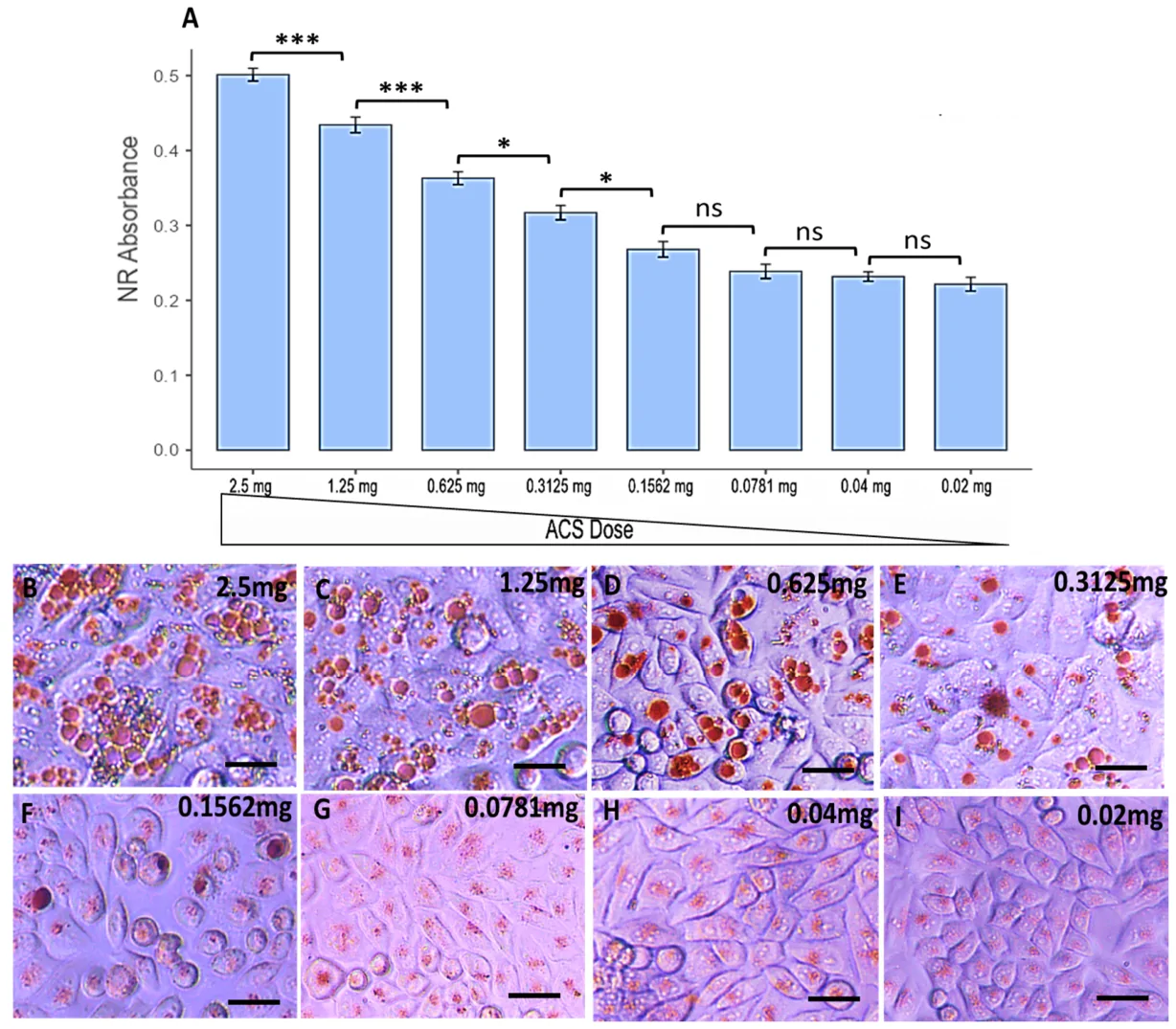
Dose-dependent induction of cytoplasmic vacuolation in ACS-treated HeLa cells. (**A**) Quantitative assessment of intracellular vacuolation using the neutral red (NR) uptake assay across increasing concentrations of activated calcium sulfate (ACS). A clear dose-dependent increase in NR accumulation is observed, reflecting progressive expansion of acidic vacuolar compartments with increasing ACS exposure. Data are presented as mean ± SD (*n* = 4). Statistical significance was determined by one-way ANOVA followed by Tukey’s multiple-comparison test. *** *p* < 0.001, * *p* < 0.05 and ns, *p* ≥ 0.05, demonstrating a consistent and concentration-dependent vacuolization response. The triangle indicates the direction of increasing ACS concentration from right to left. (**B**–**I**) Representative bright-field microscopy images of NR-stained HeLa cells treated with graded doses of ACS, illustrating the morphological progression of vacuole formation. High-dose treatment ((**B**); 2.5 mg/120 µL) results in extensive cytoplasmic vacuolation characterized by large, densely stained NR-positive vesicles occupying a substantial portion of the cytoplasm. Intermediate concentrations (**C**,**D**); 1.25 mg/60 µL and 0.625 mg/30 µL, respectively) show a marked reduction in both vacuole size and number, with fewer but clearly distinguishable acidic compartments. Further serial dilutions ((**E**,**F**,**G**); 0.3125 mg/30 µL, 0.1562 mg/30 µL, and 0.0781 mg/30 µL, respectively) reveal progressively diminished vacuolation, with smaller and more dispersed NR-positive vesicles. At the lowest concentrations ((**H**,**I**); 0.04 mg/30 µL and 0.02 mg/30 µL), cells exhibit minimal vacuolation, limited to sparse and discrete lysosome-like structures, closely resembling basal endo-lysosomal activity. Overall, these observations demonstrate a threshold-dependent and dose-responsive induction of cytoplasmic vacuolation by ACS, with both the extent and morphology of vacuoles strongly influenced by particle concentration. Scale bars: 20 µm.

At 120 µL (~2.5 mg), HeLa cells exhibited increased vacuolation, characterized by the formation of many vacuoles per cell. This phenotype was readily visualized by light microscopy and was accompanied by neutral red (NR) uptake, indicating active acidification of the vacuolar compartments (Figure 5B). At 60 µL (~1.25 mg) and at 30 µL (~0.625 mg) concentrations, vacuole formation was decreased but there was optimal distribution of vacuoles per cell, corresponding to diminished NR accumulation (Figure 5C,D).

Further reduction in particle concentration through serial dilution resulted in a pronounced attenuation of vacuolar responses. At the lowest dilution, only isolated vacuoles were detectable, reflecting minimal vacuole induction relative to higher ACS doses (Figure 5E–I). Quantitative analysis of NR uptake, presented as a bar graph with statistically analysis, corroborated these observations and demonstrated a strong positive correlation between ACS particle concentration and vacuole formation (Figure 5A).

Collectively, these findings establish particle concentration as a critical determinant of ACS-induced vacuolation. Higher particle loads promote robust vacuole formation and acidification, consistent with efficient phagocytic uptake and phagosome maturation, as evidenced by enhanced NR accumulation. In contrast, lower particle concentrations appear insufficient to trigger a sustained phagocytic response, resulting in limited vacuole formation. The serial dilution experiment further supports the existence of a threshold particle concentration required for effective vacuole induction. These results highlight the concentration dependent regulation of vacuolar dynamics in response to ACS.

**Figure 6:**
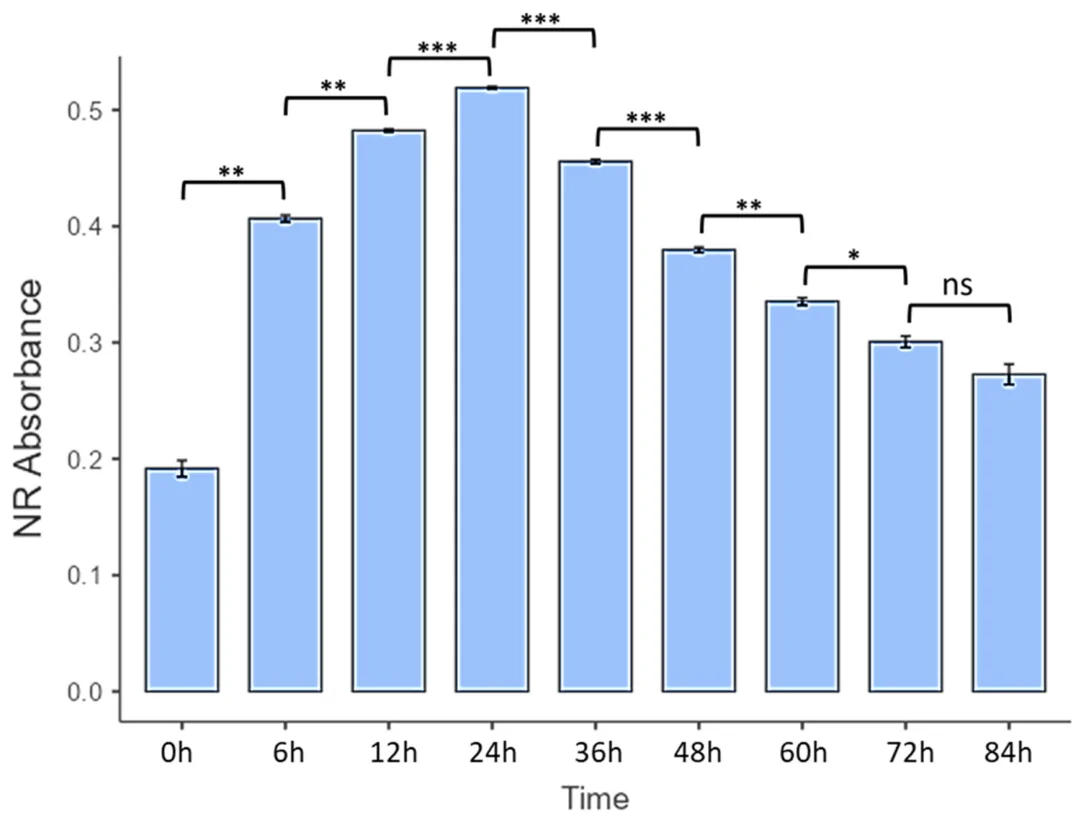
Time-dependent quantification of vacuole formation and turnover in ACS-treated cells using Neutral Red uptake assay. Neutral Red (NR) uptake was measured at indicated time points following treatment with activated calcium sulfate (ACS) particles to assess vacuole formation and intracellular processing. At 0 h, NR absorbance was minimal, indicating basal dye accumulation and absence of ACS-induced vacuoles. NR uptake increased at 6 h, reflecting early vacuole formation during ACS internalization, and further increased at 12 h, reaching a maximum at 24 h, corresponding to extensive formation of large acidic vacuoles. Subsequently, NR absorbance progressively declined from 36 h to 84 h, indicating degradation of internalized ACS particles and vacuolar turnover. NR levels at later time points (48–84 h) were lower than those observed at 6 h, consistent with active vacuolar resolution and restoration of cellular homeostasis. Data are presented as mean ± SD (*n* = 3). Because the homogeneity-of-variance assumption was violated, statistical significance was determined using Welch’s one-way ANOVA followed by Games–Howell multiple-comparison testing. * *p* < 0.05, ** *p* < 0.01, *** *p* < 0.001; ns, *p* ≥ 0.05.

Time-dependent cellular responses to activated calcium sulfate (ACS) particles were assessed by quantifying Neutral Red (NR) uptake as an indicator of vacuole formation and dynamics. At 0 h, NR absorbance was minimal, reflecting basal dye accumulation and the absence of ACS-induced vacuoles immediately after particle addition (Figure 6). By 6 h, NR uptake increased, indicating initiation of vacuole formation during early ACS internalization. NR absorbance further increased at 12 h and peaked at 24 h (Figure 6), corresponding to extensive formation of large acidic cytoplasmic vacuoles that actively accumulated NR, consistent with maximal vacuolar expansion and intracellular processing of ACS particles.

Following this peak, NR absorbance progressively declined from 36 h to 84 h, indicating degradation of internalized ACS particles within acidic vacuolar compartments and associated vacuolar turnover and recycling [47]. Notably, NR levels at 48, 60, 72, and 84 h were lower than those at 6 h (Figure 6), supporting the active vacuolar resolution and restoration of cellular homeostasis. Time-lapse video of A549 cells treated with 2 min stock sedimentation heterogeneous ACS particles demonstrated a progressive increase in vacuole number and size up to 24 h. Beyond 24 h, both vacuole number and size gradually declined. By day 4, a high degree of vacuolar turnover was observed, although a few isolated vacuoles remained visible (Video S1). This persistence is likely attributable to the heterogeneous nature of the 2 min sedimentation fraction, which contains larger ACS particles that require extended time for complete intracellular degradation. Collectively, these findings demonstrate that ACS-induced vacuolation is a dynamic and reversible process involving distinct phases of vacuole biogenesis, maturation, and recycling, which can be quantitatively monitored by NR uptake [47,49].

### 2.4. Relationship Between ACS Material Properties and Vacuolar Response

The biological response induced by activated calcium sulfate (ACS) may be influenced by its physicochemical properties, including its hydration state and particle-size heterogeneity [50]. FT-IR and XRD characterization confirmed the transformation of gypsum into an anhydrous calcium sulfate phase following thermal activation. In addition, sedimentation-based fractionation was used to address the heterogeneity of the prepared ACS suspension, and the 5 min fraction was selected for subsequent cellular experiments based on its particle distribution and reproducibility of the vacuolar response.

The selected ACS fraction induced a measurable Neutral Red (NR) response in the tested cellular models, providing a quantitative readout of the associated acidic vacuolar compartment response. These findings highlight the importance of material preparation and particle fractionation when establishing a reproducible ACS-based assay. However, the present study did not systematically vary individual physicochemical properties to establish their independent contributions to the observed cellular response. Further studies using defined particle-size fractions, additional physicochemical and molecular characterization will be required to establish a more direct relationship between ACS material properties and biological activity.

### 2.5. Suppression of ACS-Induced Vacuolation by Bafilomycin A1

**Figure 7:**
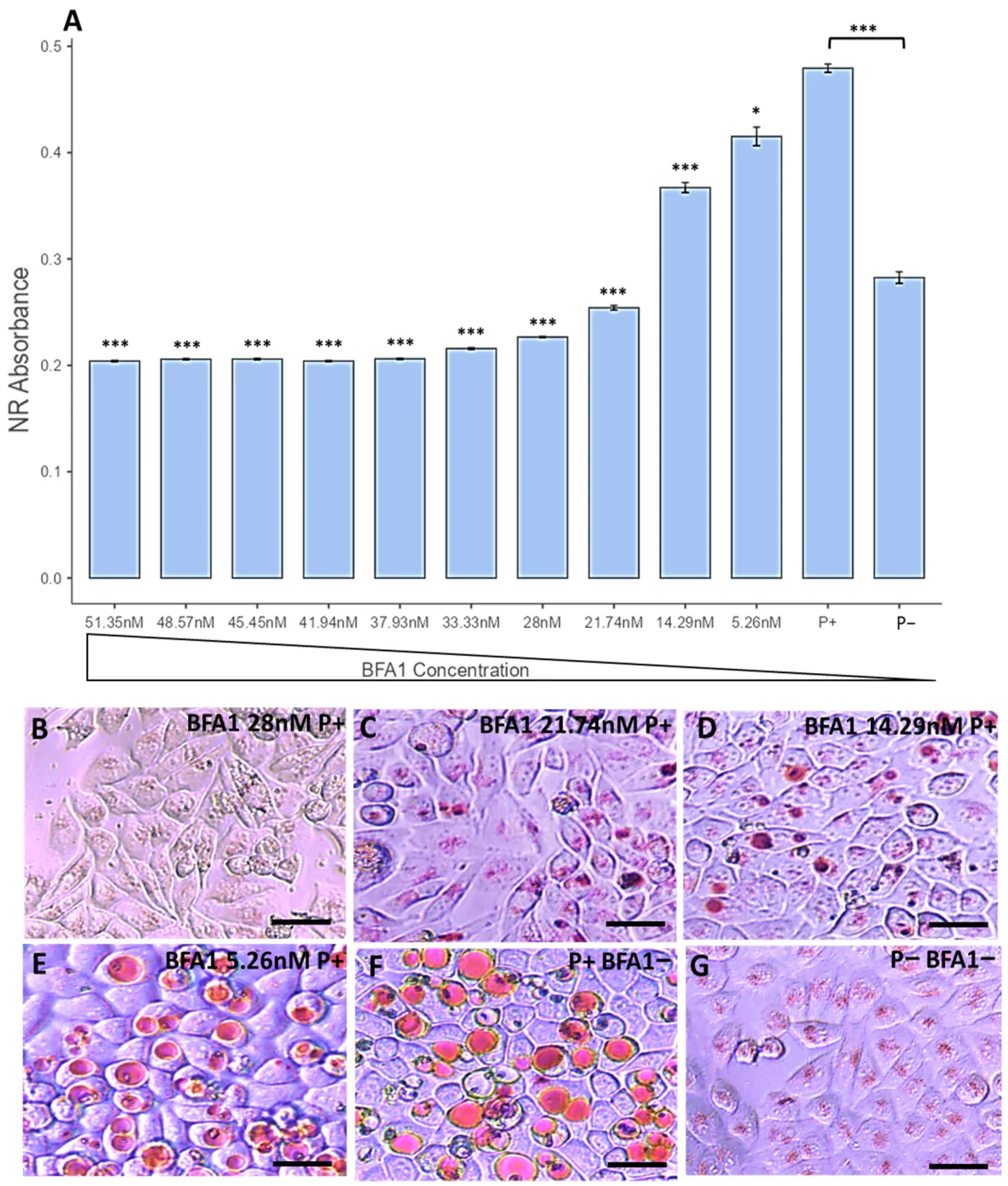
Dose-dependent inhibition of ACS-induced vacuolation by Bafilomycin A1. (**A**) Quantitative analysis of Neutral Red (NR) uptake in ACS-treated cells exposed to increasing concentrations of Bafilomycin A1. NR absorbance decreased at higher BFA1 concentrations, indicating a reduction in the NR is due to inhibition of ACS induced vacuole formation. Data are presented as mean ± SD (*n* = 4). Because the homogeneity-of-variance assumption was violated, statistical significance was determined using Welch’s one-way ANOVA followed by Games–Howell multiple-comparison testing. Comparisons were made with the ACS-treated control (P+) and the untreated control (P−). * *p* < 0.05, *** *p* < 0.001; ns, *p* ≥ 0.05. The triangle indicates the direction of increasing drug concentration from right to left. (**B**–**F**) Representative bright-field microscopy images of NR-stained cells illustrating the dose-dependent effects of Bafilomycin A1 on ACS-induced vacuolation. At lower concentrations ((**D**,**E**); 5.26–14.29 nM), cells display abundant, large cytoplasmic vacuoles with prominent NR staining. At intermediate concentrations ((**H**,**I**); 21.74–28 nM), the vacuolar phenotype is reduced, with smaller vacuoles and diminished NR staining. At higher concentrations ((**B**,**G**); 33.33–51.35 nM), vacuolation is markedly reduced, with fewer prominent vacuoles and weaker NR staining. The P+ control ((**L**); ACS-treated without BFA1) displays extensive ACS-induced vacuolation, whereas the P- control ((**M**); cells without ACS and BFA1) exhibits minimal NR staining, primarily in basal acidic compartments. Together, these observations indicate concentration-dependent inhibition of the ACS-induced vacuolar phenotype by BFA1. Scale bars: 20 µm.

The effect of bafilomycin A1 on vacuole formation was evaluated by varying its concentration in cultured cells exposed to ACS. Bafilomycin A1, known for inhibiting the vacuolar-type H^+^-ATPase (V-ATPase) [51], was used to investigate its role in vacuole formation and acidification. At higher concentrations of bafilomycin A1 (51.35 nM to 28 nM), vacuole formation was significantly inhibited (Figure 7A,B). When cells were treated with 21.74 nM of bafilomycin A1, small vacuoles began to appear, which successfully took up Neutral Red (NR) stain (Figure 7C), indicating the presence of acidic compartments. However, at lower concentrations (14.29 and 5.26 nM), the vacuoles increased dramatically in both size and number (Figure 7D,F). These larger vacuoles exhibited active NR uptake (presented as bar graphs, Figure 7A), suggesting that vacuoles formed under these conditions retained their acidic nature and likely underwent normal phagosome maturation processes.

Cell viability remained at approximately 90% even at higher Bafilomycin A1 concentrations, indicating that the concentrations used were not cytotoxic. The data suggest that bafilomycin A1 influences vacuole size and acidification in a dose-dependent manner, with lower concentrations leading to more prominent vacuole formation and acidification.

Bafilomycin A1 has a well-established role as a V-ATPase inhibitor, and its effect on vacuole acidification and maturation is well-documented in various cell types [36,37,51]. In this study, we observed that Bafilomycin A1 inhibits vacuole formation at higher concentrations, consistent with its known inhibitory action on proton pumps, which are essential for maintaining the acidic environment within phagosomes. This acidification is critical for phagosome maturation, leading to the recruitment of various hydrolases that aid in particle degradation [52]. The formation of small vacuoles at concentration (21.74 nM) of Bafilomycin A1, and their ability to take up NR, implies that V-ATPase activity was still functional, allowing partial acidification of these compartments. Interestingly, at lower concentrations (14.29 nM and below), the vacuoles not only increased in number but also in size, all actively taking up NR stain. This suggests that low doses of Bafilomycin A1 may permit vacuole formation and maturation, possibly through residual V-ATPase activity or alternative mechanisms that compensate for the partial inhibition of proton pumps.

These findings highlight the dose-dependent effects of bafilomycin A1 on phagosome dynamics and suggest that low levels of V-ATPase inhibition do not fully block vacuole formation and maturation. Furthermore, the drastic increase in vacuole size at lower concentrations points which may lead tovacuole fusion or vesicle trafficking, which may be upregulated in the absence of full V-ATPase inhibition. This insight could be useful for understanding the fine regulation of vacuole acidification and quantification and its impact on phagocytosis, particularly in macrophage dysfunction models where vacuole formation is impaired or exaggerated [51].

### 2.6. Inhibition of Vacuolar Acidification Following Bafilomycin A1 Treatment

To evaluate the effect of bafilomycin A1 (BFA1) on the acidification of pre-formed ACS-induced vacuoles, HeLa cells were first treated with 5th-minute ACS stock particles and incubated overnight to allow extensive vacuole formation. The cells were subsequently treated with 51.35 nM BFA1 and incubated for 24 or 48 h, after which neutral red (NR) and AO staining was performed to assess vacuolar acidification. ACS-treated cells without BFA1 were maintained as controls.

**Figure 8:**
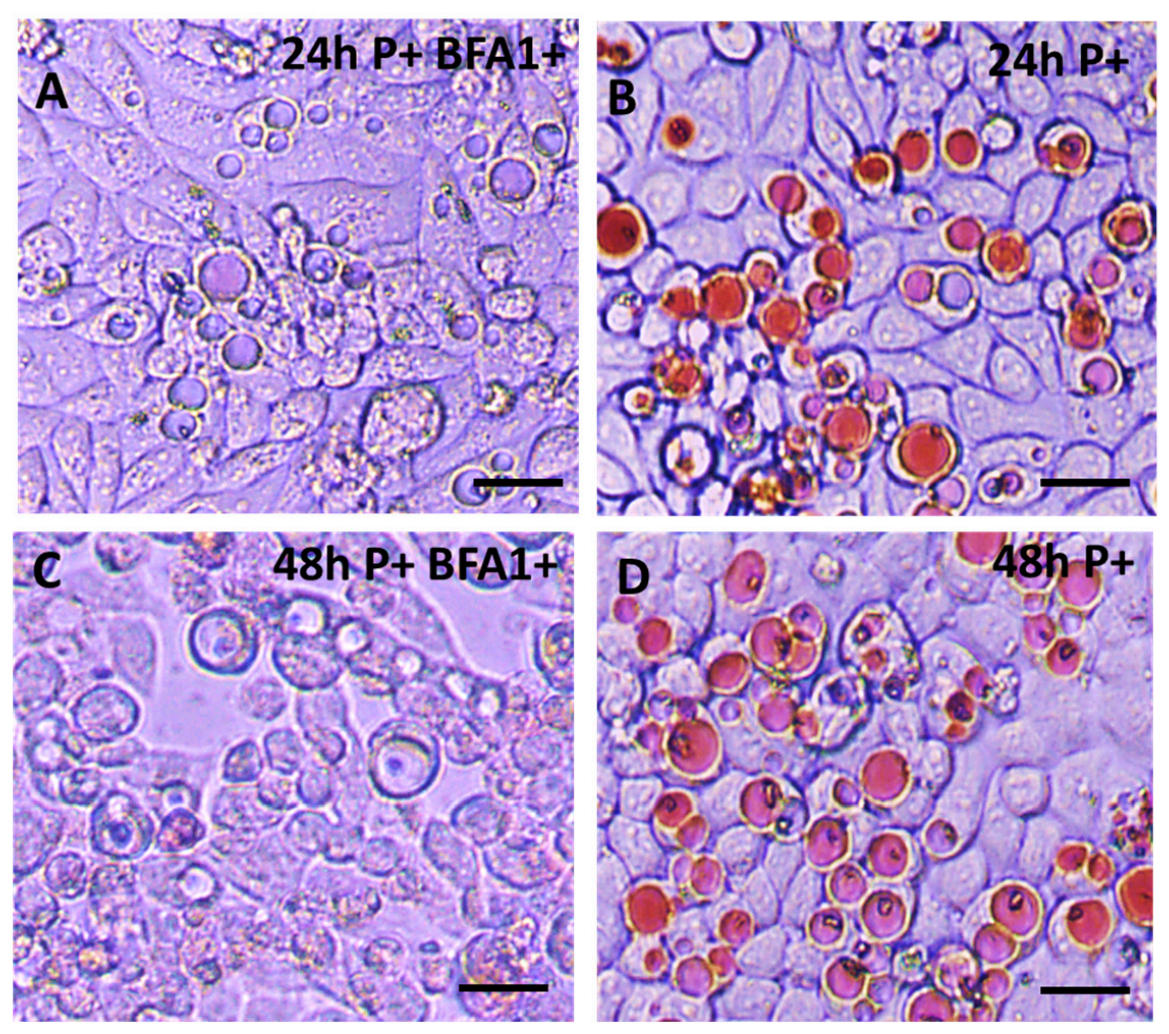
Inhibition of vacuole acidification by Bafilomycin A1. (**A**–**D**) Representative bright-field microscopy images of HeLa cells containing ACS-induced cytoplasmic vacuoles, treated with BFA1 and monitored over extended time points (24 h–48 h) using neutral red (NR) staining to assess vacuolar acidity. At 24 h post-treatment (**A**), a marked reduction in NR staining is observed, with vacuoles appearing largely unstained or faint, indicating loss of acidic luminal pH. By 48 h (**C**), NR uptake is almost completely abolished, with vacuoles either absent or present as non-acidic, translucent structures, suggesting sustained inhibition of vacuolar acidification and/or vacuole resolution. Corresponding time-matched control conditions ((**B**,**D**); 24 h and 48 h) display robust NR-positive vacuoles with strong staining intensity, confirming that vacuolar acidification remains intact in the absence of BFA1.

Following 24 h of BFA1 treatment, ACS-induced vacuoles showed a marked reduction in NR uptake compared with ACS-treated control cells, indicating a substantial reduction in vacuolar acidification. This inhibitory effect was maintained after 48 h of BFA1 treatment, with vacuoles showing little or no NR accumulation compared with the corresponding ACS-treated controls (Figure 8). These findings demonstrate that BFA1 effectively inhibits the acidification of established ACS-induced vacuoles following prolonged exposure.

The observed reduction in NR uptake following BFA1 treatment is consistent with inhibition of vacuolar H^+^-ATPase (V-ATPase), which is responsible for maintaining proton accumulation within acidic intracellular compartments. Because the vacuoles were already established before BFA1 treatment, the results indicate that continued acidification of pre-formed ACS-induced vacuoles depends on V-ATPase activity. The persistence of reduced NR uptake at 48 h further suggests sustained impairment of vacuolar acidification following prolonged V-ATPase inhibition [51].

**Figure 9:**
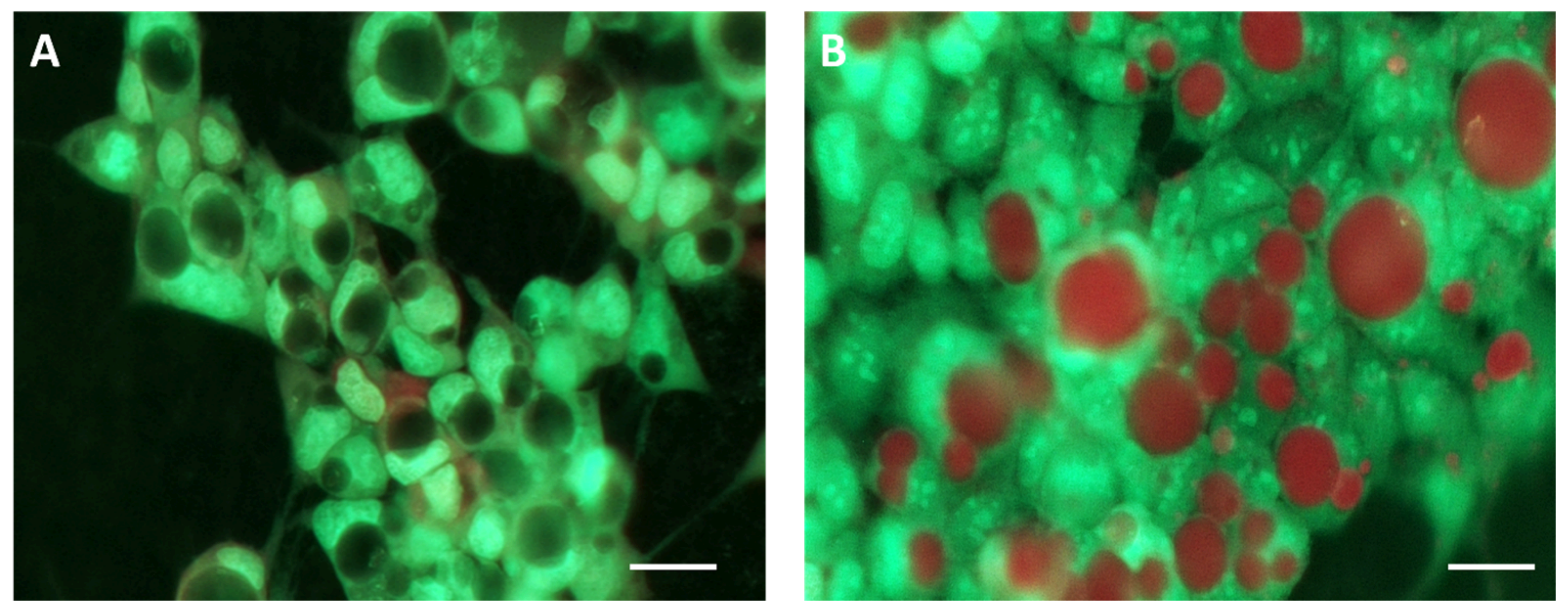
Acridine orange assay demonstrating inhibition of vacuolar acidification by Bafilomycin A1. (**A**) Fluorescence microscopy images of ACS-treated cells exposed to Bafilomycin A1 showing vacuoles that failed to accumulate acridine orange, appearing colourless, while nuclei fluoresced green. (**B**) Control cells exhibited strong orange-red fluorescence within vacuoles, confirming acidic compartments, along with green nuclear staining. These results indicate that Bafilomycin A1 selectively blocks vacuolar acidification without altering nuclear staining (Scale = 15 µm).

This was further confirmed by Acridine Orange staining, where ACS-treated control cells exhibited prominent orange–red fluorescence within vacuoles, whereas BFA1-treated cells showed little or no vacuolar orange–red fluorescence, while nuclear green fluorescence was retained (Figure 9A,B). These findings demonstrate that prolonged BFA1 treatment effectively disrupts the acidification of established ACS-induced vacuoles, consistent with inhibition of V-ATPase-dependent proton accumulation [51].

This was further confirmed by Acridine Orange staining, where ACS-treated control cells exhibited prominent orange–red fluorescence within vacuoles, whereas BFA1-treated cells showed little or no vacuolar orange–red fluorescence, while nuclear green fluorescence was retained (Figure 8A,B). These findings demonstrate that prolonged BFA1 treatment effectively disrupts the acidification of established ACS-induced vacuoles, consistent with inhibition of V-ATPase-dependent proton accumulation [51].

Overall, these findings demonstrate that BFA1 disrupts the acidic environment of established ACS-induced vacuoles and support the involvement of V-ATPase-dependent acidification in the maintenance and maturation of these vacuolar compartments.

### 2.7. Pharmacological Modulation of ACS-Induced Vacuolation

**Figure 10:**
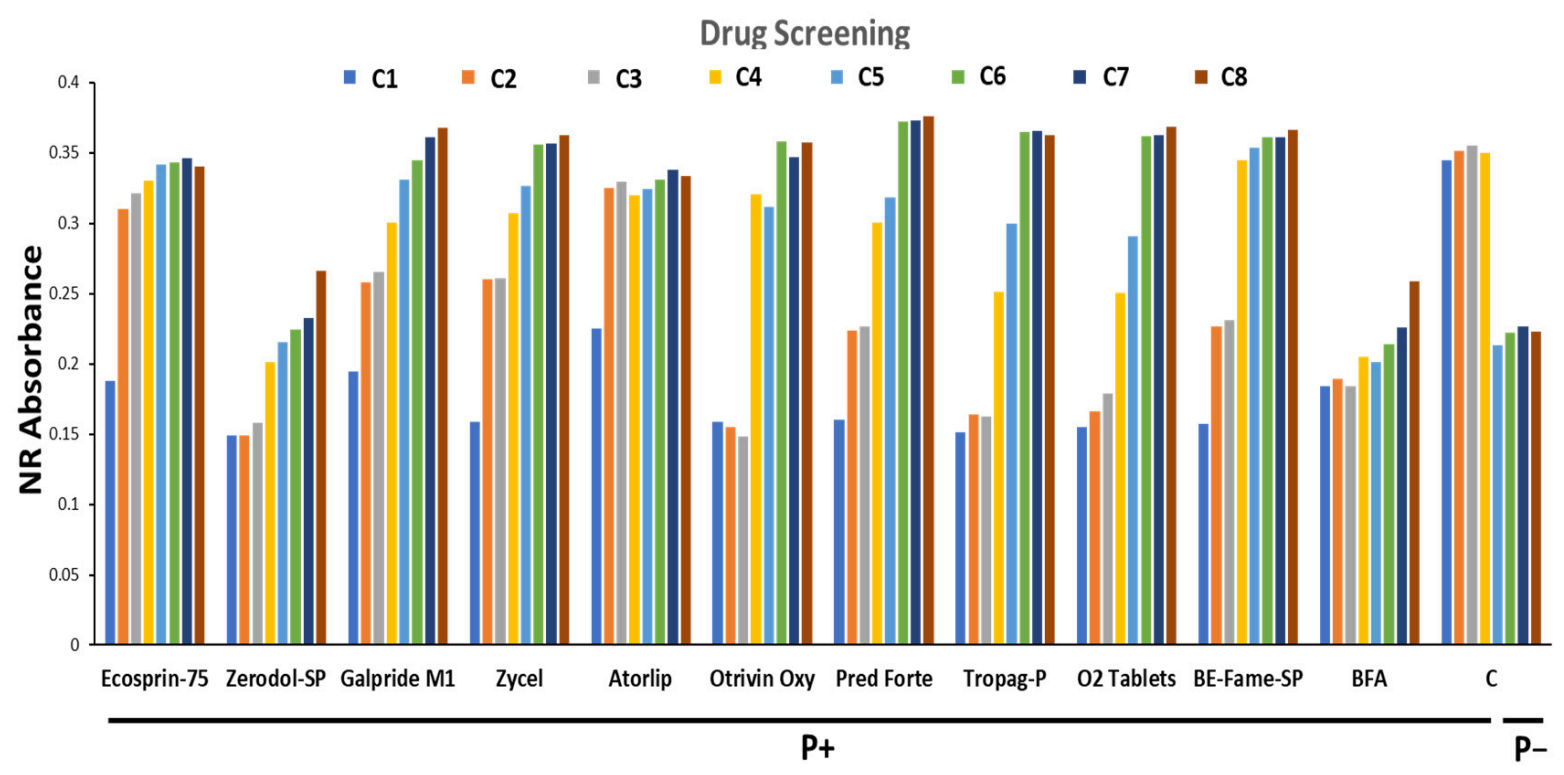
Neutral Red (NR)–based quantification of vacuole formation in ACS-treated HeLa cells under drug screening conditions. HeLa cells were treated with activated calcium sulfate (ACS) in the presence or absence of 10 drugs across a serial dilution (C1, the highest concentration, to C8, the lowest). The control (ACS alone, i.e., P+) displayed strong NR uptake, confirming robust vacuole induction, while the control (untreated cells i.e., P−) exhibited minimal NR staining, representing basal lysosomal activity. Drug-specific responses were observed: Drug Ecosprin-75, Atorlip, and Otrivin Oxy showed no inhibition, maintaining NR uptake similar to ACS control; Zerodol-Sp caused marked suppression of NR uptake at higher concentrations, indicative of cytotoxicity, with partial recovery at lower doses; Galpride-M1 showed graded inhibition at the highest concentration followed by progressive restoration of vacuole formation; Drugs Pred Forte, Torpag-P, O2 and BE-Fame-sp induced cytotoxicity at higher concentrations with subsequent recovery at lower doses, as evidenced by restored NR accumulation. Data highlight the ability of ACS-induced vacuolation, quantified by NR staining, to discriminate between cytotoxic suppression and direct inhibition of vacuole biogenesis, establishing this assay as a functional platform for drug screening. The drug screening data represent preliminary proof-of-concept observations from a single screening experiment (*n* = 1); therefore, comparisons between drug-treated groups and P+/P− (*n* = 4 each) controls are descriptive and no inferential statistical testing or significance testing was not performed.

Neutral Red (NR) staining was employed to quantitatively assess vacuolar accumulation in HeLa cells treated with activated calcium sulfate (ACS) in the presence of ten different drugs (Figure 9). HeLa cells were seeded in 96-well plates and allowed to adhere overnight. For drug screening, columns 1–10 were assigned to individual drugs, each tested as an eight-point serial dilution, followed by uniform addition of 100 µL freshly prepared 5 min ACS suspension to all wells. Column 11 contained BFA1 positive controls (5.26–51.35 nM), while column 12 included ACS-only controls and untreated cell controls. After 24 h of culture, ACS-treated control cells exhibited robust NR uptake, confirming consistent vacuole induction by ACS, whereas untreated cells displayed minimal NR accumulation, reflecting basal lysosomal activity (Figure 10).

Distinct drug-specific response patterns were observed across the concentration gradient (wells 1–8). Drug 1 showed no inhibitory effect on vacuole formation, with NR uptake closely matching ACS-treated controls and exceeding the lowest concentration of the drug-positive BFA1 control, indicating that vacuole biogenesis remained unaffected (Figure 10). In contrast, Drug 2 exhibited strong inhibition at higher concentrations, with the first three concentrations yielding NR values lower than the untreated control and below the highest concentration of the BFA1 control, indicative of pronounced drug-induced cytotoxicity (Figure 10). From the fourth concentration onward, NR uptake gradually increased and approached the levels observed in untreated control and high-dose BFA1 control cells, indicating recovery of cell viability and partial restoration of vacuole formation (Figure 10).

Drug 3 and 4 demonstrated transient inhibition at the first concentration, followed by dose-dependent recovery, with vacuole formation restored from the second concentration onward and ultimately reaching levels comparable to ACS-treated controls (Figure 10). Drug 5 showed no measurable inhibition of vacuolation, maintaining robust NR uptake similar to ACS-treated cells and exceeding the lowest concentration of the BFA1 control (Figure 10).

Drug 6 induced marked cytotoxicity at high concentrations (1st–3rd), as reflected by reduced NR uptake, followed by recovery at lower concentrations (≥4th), where vacuole formation resumed, evidenced by a progressive increase in NR accumulation (Figure 10). A comparable trend was observed for Drug 7, wherein the first concentration caused cell death, the second and third concentrations supported cell viability with suppressed vacuolation, and from the fourth concentration onward, vacuole formation was restored (Figure 10). Drugs 8, 9, and 10 exhibited response patterns analogous to Drugs 6 and 7, indicating early concentration-dependent cytotoxicity followed by recovery and re-establishment of vacuolar activity at lower concentrations (Figure 10).

Collectively, these results underscore the dual utility of ACS-induced vacuolation as both a sensitive readout of particle–cell interactions and a functional in vitro platform for drug screening. Drugs 2, 6, 7, 8, 9, and 10 displayed concentration-dependent cytotoxic effects at higher doses, followed by recovery and modulation of vacuole formation at lower concentrations. In contrast, Drugs 1 and 5 exerted negligible effects on vacuolar dynamics, suggesting limited involvement in phagocytosis-associated pathways. Drug 3 and 4 exhibited a graded inhibition–recovery profile, highlighting its potential role as a modulator of vacuolar dynamics rather than a purely cytotoxic agent.

For interpretation of the drug-screening results, NR absorbance was compared with the ACS-treated (P+) and ACS-untreated (P−) controls. NR absorbance below the P− level was considered indicative of drug-induced cytotoxicity. Absorbance equal to or greater than P− but lower than P+ indicated that the cells remained viable but exhibited inhibition of ACS-induced vacuole formation. In contrast, absorbance equal to or greater than P+ indicated retention or restoration of ACS-induced vacuole formation. These responses were generally concentration-dependent, with cytotoxicity or vacuole inhibition predominantly observed at higher drug concentrations, whereas lower concentrations showed retention/restoration of vacuole formation.

**Figure 11:**
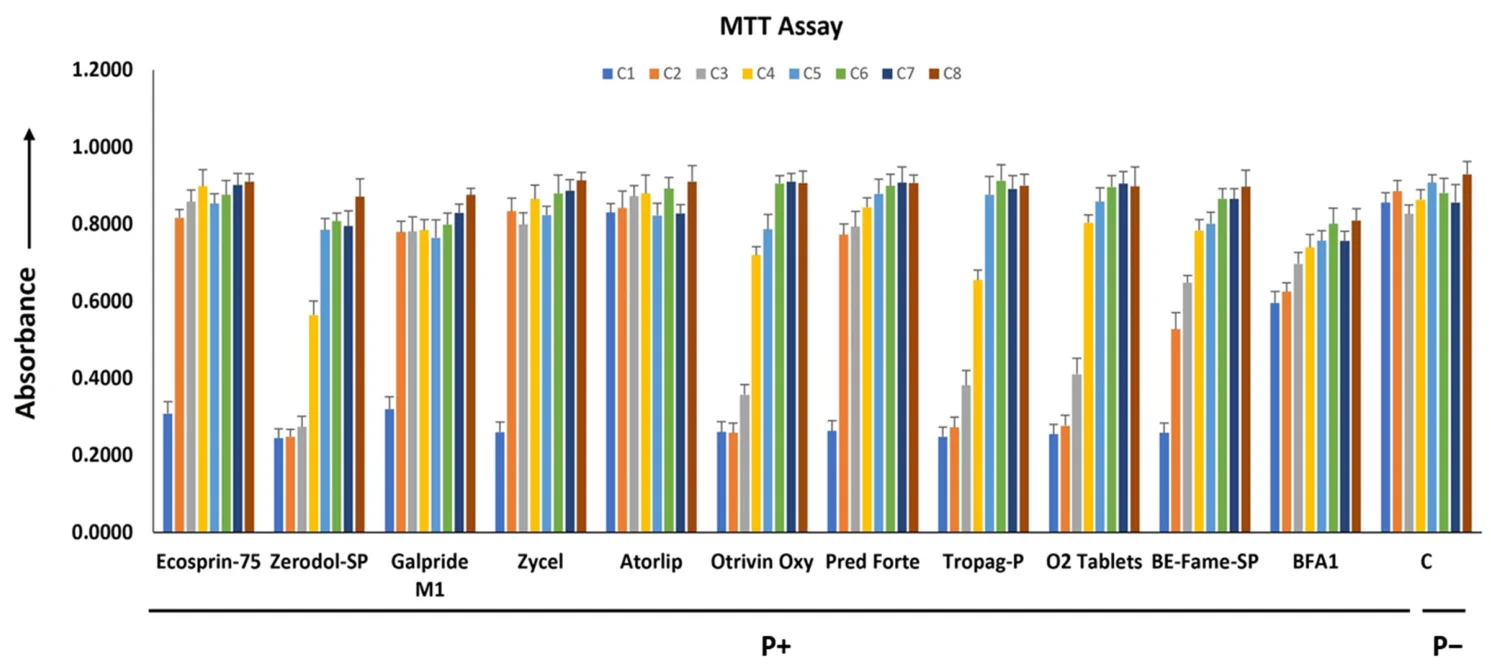
MTT assay for assessment of cytotoxicity following treatment with pharmaceutical formulations. HeLa cells were exposed to serial dilutions of the selected pharmaceutical formulations for 24 h, and cell viability was assessed using the MTT assay. C1 represents the highest drug concentration and C8 the lowest. Possible cytotoxicity was observed at higher concentrations (C1–C3) of Zerodol-SP, Otrivin Oxy, Tropag-P, O2 tablets, and BE-Fame-SP, with no apparent cytotoxicity at lower concentrations (C5–C8). Ecosprin-75, Galpride-M1, Zycel, and Pred Forte showed possible cytotoxicity primarily at C1, whereas Atorlip showed no clear evidence of cytotoxicity across the tested concentrations. For control C, C1–C4 represent the control P+ (ACS-treated cells), while C5–C8 represent the control P− (cells without ACS treatment). Both controls showed broadly comparable MTT responses despite the difference in ACS-induced vacuolation. Furthermore, drug concentrations that inhibited ACS-induced vacuolation in the neutral red (NR) assay did not consistently show cytotoxicity in the MTT assay. The results are presented as the mean ± SD (*n* = 3).

An MTT assay was performed under the same conditions as the drug screening to complement the ACS-NR results and to separate drug-induced cytotoxicity from specific inhibition of ACS-induced vacuolation. The assay showed concentration-dependent reductions in cell viability for several pharmaceutical formulations (Figure 11). Zerodol-SP, Otrivin Oxy, Tropag-P, O2 tablets, and BE-Fame-SP markedly reduced viability at the higher concentrations (C1-C3), with clear recovery at lower concentrations. Ecosprin-75, Galpride-M1, Zycel, and Pred Forte reduced viability mainly at the highest concentration (C1), whereas Atorlip showed no clear concentration-dependent cytotoxicity. The P+ (ACS-treated) and P− (untreated) controls gave broadly comparable MTT responses, indicating that ACS itself does not impair cell viability, even though vacuolation occurs only in P+ cells (Figure 11) [46,47].

The MTT and NR data were interpreted together, using the P− control as the reference for drug-induced cytotoxicity. Concentrations at which MTT values fell below the P− level were considered potentially cytotoxic, whereas values equal to or above the P− level indicated no apparent cytotoxicity. Where NR uptake was reduced but MTT viability was maintained, the reduction was attributed to inhibition of ACS-induced vacuole formation rather than cell death. This combined analysis showed that the loss of NR signal at the highest drug concentrations largely reflects cytotoxicity, whereas the NR changes at lower, non-cytotoxic concentrations reflect modulation of vacuolation.

The ACS-induced vacuolation assay, coupled with Neutral Red (NR)–based quantification, establishes a robust and functionally informative in vitro platform for screening modulators of phagocytosis-associated vacuole biogenesis [47]. The reproducible formation of large, acidified vacuoles in ACS-treated cells generates a high-contrast, quantifiable readout that sensitively captures drug-induced perturbations in vacuolar dynamics across a concentration gradient [39]. This system effectively discriminates between compounds that exert direct inhibitory effects on vacuole formation and those that suppress NR accumulation secondary to cytotoxicity, as evidenced by distinct inhibition–recovery profiles observed among the tested drugs. Importantly, the graded responses—ranging from complete resistance to vacuole inhibition, transient suppression with recovery, and concentration-dependent cytotoxicity followed by restoration of vacuolar activity—underscore the assay’s ability to resolve mechanistically diverse drug actions within a single experimental framework. Unlike conventional phagocytosis assays that rely on fluorescent labelling, end-point imaging, or specialized instrumentation [53], the ACS–NR platform offers a simple, low-cost, and scalable approach compatible with 96-well formats, making it well suited for preliminary drug screening. By directly linking particle-induced vacuolation with cellular viability and lysosomal acidification, this platform provides a physiologically relevant functional readout for identifying candidate inhibitors or modulators of phagocytic and vacuolar pathways, thereby offering clear advantages for early-stage screening of compounds targeting phagocytosis-related processes.

From a drug-screening perspective, the ACS-induced vacuolar system provides a quantitative platform for identifying compounds that interfere with vacuolar acidification and maturation. The established BFA1 response demonstrates that loss of vacuolar acidification can be detected using the ACS–NR assay, providing a pharmacological reference for screening. Using this platform, we have screened a panel of commercially available drugs and identified multiple compounds that inhibit the ACS-induced vacuolar response. However, specific inhibitors of vacuolar acidification and maturation have not yet been identified. We are therefore continuing to screen additional commercially available drugs for their ability to inhibit vacuolar acidification and maturation. If such compounds are identified, their effects on vacuolar maturation will be characterized and reported in future studies.

Collectively, these observations demonstrate that prolonged exposure of BFA1, results in a progressive and ultimately complete loss of vacuolar acidification, consistent with sustained inhibition of V-ATPase activity and impaired maintenance of proton gradients within vacuolar compartments. Scale bars: 20 µm.

## 3. Materials and Methods

### 3.1. Preparation of Activated Calcium Sulfate Particles

Raw gypsum (CaSO_4_·2H_2_O) was coarsely powdered and transferred to a ceramic vial. The material was thermally activated by calcination in a muffle furnace at 700 °C for 1 h, followed by passive cooling to room temperature. The resulting activated calcium sulfate (ACS, CaSO_4_) was stored in a borosilicate glass vial under dry conditions for subsequent experiments.

### 3.2. Characterization of Activated Calcium Sulfate Particles

Fourier-transform infrared (FT-IR) spectroscopy was performed to assess structural differences between gypsum and activated calcium sulfate (ACS). Spectra were acquired at room temperature using an AGILENT CARY 630 (Agilent Technologies, Santa Clara, CA, USA) FT-IR spectrometer equipped with an attenuated total reflectance (ATR) module and operated via MicroLab PC software version 5.3.1748. Measurements were conducted in triplicate using three independent samples per condition to ensure reproducibility.

Powder X-ray diffraction (XRD) analysis was carried out on gypsum and ACS samples using a high-resolution X-ray diffractometer (PANalytical High-Resolution XRD-I, PW 3040/60, Philips, San Jose, CA, USA) equipped with a Cu Kα radiation source (λ = 1.5406 Å). Diffraction patterns were collected over a 2θ range of 10–80° with a step size of 0.02° and a dwell time of 1 s. The X-ray generator was operated at 40 kV and 30 mA. Phase identification was performed using the JCPDS database, and crystallite size was estimated using the Scherrer equation. Peak fitting and background subtraction were carried out using X’Pert^®^ analysis software, version 5.3.

### 3.3. Preparation of Activated Calcium Sulfate Suspensions and Sedimentation-Based Fractionation

Activated calcium sulfate (ACS) suspensions were prepared by dispersing 100 mg of ACS in 7 mL of Dulbecco’s Modified Eagle Medium (DMEM) supplemented with 10% fetal bovine serum (FBS) in 9 separate sterile 15 mL centrifuge tubes. Each suspension was vortexed to achieve uniform particle dispersion.

At every 1 min intervals, 5 mL aliquots were carefully aspirated from the upper phase of each suspension and transferred to fresh tubes. This stepwise sedimentation procedure was continued up to 9 min, yielding nine distinct ACS fractions corresponding to sedimentation times of 1–9 min. Each sedimentation fractions was gently pipetted to achieve uniform particle dispersion, and each tube was treated as an independent stock for time-dependent sedimentation analysis. From each fraction, 30 µL of suspension was mounted on glass slides, dried and examined using an inverted microscope (EA PRIME Inverted) to evaluate particle size distribution and the progression from heterogeneity to relative homogeneity (*n* = 3).

### 3.4. Cell Culture and Seeding

Dulbecco’s Modified Eagle Medium (DMEM), fetal bovine serum (FBS), antibiotics, and Trypsin–EDTA were purchased from Thermo Fisher Scientific, Waltham, MA, USA. HeLa, 3T3-L1, SH-SY5Y, and RAW 264.7 cell lines were obtained from the National Centre for Cell Science (NCCS), Pune, India. Cells were maintained in DMEM supplemented with 10% heat-inactivated FBS, 100 µg/mL penicillin, 100 µg/mL streptomycin, and 5 µg/mL amphotericin B under standard culture conditions (37 °C, 5% CO_2_, and 95% relative humidity).

Stock cultures were grown in 25 cm^2^ tissue culture flasks and sub-cultured using Trypsin–EDTA. Briefly, cells were washed twice with 1x phosphate-buffered saline (PBS), detached with 500 µL Trypsin–EDTA, and neutralized with 1 mL DMEM. The resulting cell suspension was gently pipetted to ensure uniform dissociation, transferred to a 15 mL centrifuge tube, and centrifuged at 1000 rpm for 5 min. The supernatant was carefully discarded, and the cell pellet was re-suspended in DMEM to a final volume of 10 mL. For experiments, cells were seeded into 96-well plates by dispensing 90 µL of the cell suspension (10,000 cells) per well and incubated overnight under standard culture conditions to achieve approximately 70% confluency prior to treatment.

### 3.5. Vacuole Induction and Quantification

Vacuole induction experiments were performed 24 h after cell seeding in 96-well plates. For treatment, 30 µL of each activated calcium sulfate (ACS) stock sedimentation fraction (1–9 min) was resuspended by gentle pipetting and added to individual wells containing pre-seeded HeLa cells (*n* = 3). Following overnight incubation under standard culture conditions, cells were examined using a bright-field inverted microscope and subsequently subjected to neutral red (NR) staining for vacuole quantification.

For comparative analysis, the 5 min sedimentation fraction, representing a relatively homogeneous population enriched in smaller ACS particles, was gently resuspended, and 100 µL of the fraction was added to wells containing pre-seeded HeLa, 3T3-L1, SH-SY5Y, and RAW 264.7 cells (*n* = 3). Cells were incubated overnight and processed for NR staining to assess vacuole formation. ACS-treated cells served as the particle-treated control, whereas cells cultured without ACS served as the untreated control. Quantitative NR absorbance data were statistically analyzed to evaluate the relationship between ACS particle exposure and vacuole formation.

### 3.6. Dose- and Time-Dependent Response of Activated Calcium Sulfate Particles

To evaluate dose-dependent cellular responses, the 5 min sedimentation fraction of activated calcium sulfate (ACS) was used for the experiment. The particle–medium suspension was re-suspended by thorough pipetting prior to dilution to ensure homogeneity.

Serial dilutions of the ACS 5 min sedimentation fraction were prepared to generate graded particle concentrations. For the initial dose range, aliquots of 120 µL, 60 µL, and 30 µL of the small-particle suspension were transferred into separate 1.5 mL microcentrifuge tubes (*n* = 3 per dose), dried, and weighed independently to determine particle mass. In parallel, corresponding volumes of DMEM (120, 60, and 30 µL) were transferred into separate tubes, dried, and weighed (*n* = 3) and used as volume-matched control masses for the respective ACS aliquots. It was determined that 120 µL, 60 µL, and 30 µL of the suspension contained approximately 2.5 mg, 1.25 mg, and 0.625 mg, respectively. The corresponding aliquots of 120 µL, 60 µL, and 30 µL from the 5 min sedimentation fraction were added to individual wells of a 96-well plate containing pre-seeded HeLa cells, representing high, medium, and low particle doses, respectively.

To assess vacuolation at lower particle concentrations, further stepwise serial dilutions were performed. Briefly, 30 µL of the small-particle suspension was mixed with 30 µL DMEM, and 30 µL of this mixture was added to a well containing HeLa cells. The remaining suspension was sequentially diluted by the addition of 30 µL DMEM, and 30 µL from each dilution was transferred to successive wells, thereby generating progressively lower particle concentrations. Following ACS exposure, cells were incubated overnight under standard culture conditions (*n* = 3). Vacuole formation was assessed by neutral red (NR) staining, and stained cells were examined by microscopy. NR uptake was quantified, and the resulting data were analyzed and presented as bar graphs to depict the relationship between ACS particle dose and vacuolation.

To evaluate time-dependent responses, the 5 min stock sedimentation fraction of ACS was used. The particle–medium suspension was resuspended, and subsequently, 100 µL of the fraction containing smaller particles was added to the wells of a 96-well plate pre-seeded with HeLa cells. Neutral Red (NR) staining was performed to assess vacuolation at defined time points. Briefly, 60 µL of Neutral Red solution was added at 0, 6, 12, 24, 36, 48, 60, 72, and 84 h post-treatment (*n* = 3 per time point), and the resulting data were statistically analyzed. For comparative analysis, time-lapse imaging was performed to assess time-dependent cellular responses in A549 cells treated with 2 min stock-sedimented ACS particles.

### 3.7. Dose-Dependent Vacuole Inhibition Using Bafilomycin A1

Bafilomycin A1 (100 µg; MedChemExpress, NY, USA) was used to evaluate dose-dependent inhibition of vacuole formation. A stock solution of Bafilomycin A1 (BFA1) at a concentration of 100 µg/mL (160.5 µM) was prepared by dissolving 100 µg of BFA1 in 50 µL of DMSO, followed by dilution in 950 µL of DMEM. 1 µL of the stock solution was diluted in 1.6 mL of DMEM to obtain a working solution of 100 nM. The solution was mixed thoroughly by repeated pipetting to ensure uniform distribution.

Dose-dependent distribution of Bafilomycin A1 was performed by adding 95 µL to 5 µL of the 1.6 mL working solution to wells of a 96-well plate pre-seeded with HeLa cells. Following Bafilomycin A1 exposure, 100 µL of 5 min stock sedimentation fraction of ACS suspension was added to each well. Cells were then incubated overnight under standard culture conditions to induce vacuole formation (*n* = 3).

After incubation, cells were stained with neutral red (NR) and examined to determine the concentration of BFA1 at which vacuole formation was first observed. NR uptake by vacuoles was quantified, and the data were statistically analyzed to illustrate the correlation between Bafilomycin A1 concentration and vacuole formation.

### 3.8. Inhibition of Vacuole Acidification by Bafilomycin A1

To evaluate the effect of BFA1 on vacuole dynamics, HeLa cells were seeded in 96-well plates and treated with 100 µL of 5th-minute ACS stock particles to induce extensive vacuole formation. Cells were incubated overnight under standard culture conditions to allow vacuole formation. Following vacuole formation, 95 µL of BFA1 working solution (51.35 nM) was added, and the cells were subsequently incubated for 24 and 48 h. After 24 and 48 h of incubation with BFA1, neutral red (NR) staining was performed to assess vacuolar acidity. ACS-treated cells without BFA1 were maintained as controls and subjected to NR staining at the corresponding time points.

### 3.9. Neutral Red Staining

Relative vacuolation was measured in mammalian cells using the Neutral Red dye [29,49]. The experiments took place in 96-well plates. Neutral red (0.5 mg/mL) was added to each well, incubated for 30 min in a CO_2_ incubator, washed two times with 1x PBS, and eluted with a destaining solution (100 µL of 50% dehydrated ethanol, 49% deionized water, and 1% glacial acetic acid). Neutral Red absorption was monitored by measuring the absorbance at 492 nm with an EnVision Multimode plate reader (Perkin Elmer, Hopkinton, MA, USA).

### 3.10. Acridine Orange Uptake Assay

3T3-L1 Cells were cultured on cavity slides by adding 100 µL of cell suspension to two cavities per slide, leaving the middle cavity empty to prevent cross-contamination. Each cavity was supplemented with 50 µL of Dulbecco’s Modified Eagle Medium (DMEM) and incubated at 37 °C in a humidified atmosphere containing 5% CO_2_ for 24 h to facilitate cell adherence and stabilization.

Cells were treated with 5 min sedimentation fraction of ACS to induce vacuole formation and incubated for an additional 24 h under standard experimental conditions. To evaluate the effect of bafilomycin A1 on vacuolar dynamics, 95 µL of bafilomycin A1 working solution was added to one cavity, while the second cavity served as an untreated control. Both cavities were incubated under the same conditions for another 24 h. Following incubation, cells were stained with acridine orange solution (1 mg/mL) to selectively label vacuoles and acidic vesicular organelles. The staining was performed by incubating the cells with the dye for 15 min at 37 °C. Post-staining, the cells were washed thrice with sterile phosphate-buffered saline (PBS) to remove excess dye and reduce nonspecific background fluorescence. The stained slides were immediately examined using a fluorescence microscope (DiGi 110 Infinity) to assess cellular morphology and acridine orange uptake, enabling the characterization of vacuolar acidification and dynamics.

### 3.11. Drug Screening

An in vitro drug screening assay was developed to identify inhibitors of phagocytosis-associated vacuole formation. Bafilomycin A1–based vacuolar inhibition was used as a positive control. Commercially available pharmaceutical formulations were used for drug preparation. For solid formulations, tablets containing ≤5 mg of active ingredient were processed using two tablets, whereas tablets containing >5 mg of active ingredient were processed using a single tablet. Tablets were transferred to sterile 15 mL centrifuge tubes, resuspended in 7 mL sterile phosphate-buffered saline (PBS), and vortexed until complete dispersion or solubilization. The suspensions were centrifuged at 3000 rpm for 10 min to pellet insoluble excipients, and the clarified supernatant was collected and stored at 4 °C until further use. Liquid drug formulations were used directly, and 100 µL of the undiluted preparation was taken as the stock solution.

For drug screening experiments, columns 1–10 of a 96-well plate were used to evaluate 10 different drugs (Table S1), with each compound serially diluted across wells within its respective column. Column 11 served as the drug-positive control; the first four wells received 51.35, 48.57, 45.45, and 41.9 nM of BFA1 working solution, respectively, while the remaining four wells received 28, 21.74, 14.29, and 5.26 nM of the BFA1 working solution, in accordance with the dose-dependent vacuole inhibition protocol described above.

Column 12 served as the control column, with the first four wells receiving 5 min sedimentation fraction of ACS suspension alone (without drug treatment) and the remaining wells containing untreated cells without ACS or drugs.

Serial dilutions of each drug stock were prepared in DMEM to generate a concentration gradient. Pre-existing media was removed and, briefly, 100 µL of the drug stock solution was mixed with 100 µL DMEM, and 100 µL of the resulting dilution was added to the first well of a 96-well plate pre-seeded with HeLa cells. Subsequent dilutions were generated by sequential addition of 100 µL DMEM to the remaining solution, thorough mixing, and transfer of 100 µL to successive wells. This process was repeated across eight wells to obtain eight drug concentrations, with a final volume of 100 µL per well.

Following drug treatment, vacuole formation was induced by the addition of 100 µL of the fifth-minute homogenized ACS particle suspension to each well. Plates were incubated at 37 °C in a humidified atmosphere containing 5% CO_2_ for 24 h. Neutral red (NR) dye (60 µL) was then added directly to each well and incubated for 15 min at 37 °C. Cells were washed twice with PBS to remove excess, non-internalized dye. Vacuolar NR uptake was visualized using a bright-field inverted microscope and quantified using a microplate reader. Quantitative data were analyzed, and dose-dependent effects of drug treatment on vacuolar NR uptake were represented graphically.

Commercial pharmaceutical formulations were used for the initial proof-of-concept drug-repurposing screen because these drugs are established clinically used products whose marketed formulations have undergone extensive safety and toxicity evaluation. The objective is to determine whether existing drugs could modulate ACS-induced vacuole formation. Tablet formulations were processed to remove insoluble particulate material before application to cells; therefore, the resulting screening hits were considered candidates for vacuolar inhibition.

### 3.12. MTT Assay

Cell viability was assessed by MTT assay in HeLa cells in the presence of ACS. Cells were seeded at 7500 cells/well in 96-well plates and incubated for 24 h. Cells were then treated with serial dilutions (eight concentrations) of the ten pharmaceutical formulations, prepared as described in Section 3.11 and starting from the highest concentrations listed in Table S1, or with BFA1 (51.35, 48.57, 45.45, 41.9, 28, 21.74, 14.29, and 5.26 nM), followed by addition of 100 µL of the 5 min ACS fraction, as in the vacuolation assay. Cells were incubated for 24 h at 37 °C in 5% CO_2_. P+ denotes ACS-treated cells (with or without drug), and P− denotes untreated control cells without ACS or drug. After treatment, the medium was completely removed and replaced with fresh medium (100 µL/well). MTT (Thiazolyl Blue Tetrazolium Bromide; Thermo Fisher Scientific, MA, USA; 10 µL of 5 mg/mL) was added to each well (final concentration approximately 0.45 mg/mL) and incubated for 3 h at 37 °C. The MTT-containing medium was carefully removed, and 100 µL DMSO (HiMedia, India) was added to dissolve the formazan crystals. After 1 h of incubation, absorbance was measured at 540 nm using an ELISA plate reader (ER-181s). Data are presented as mean ± SD (*n* = 3).

### 3.13. Statistical Analysis

Data are presented as mean ± standard deviation (SD). For experiments comparing ACS treatment across different sedimentation times and across different cell types (Figure 4), two-way analysis of variance (ANOVA) was performed with the respective experimental factors and their interaction included in the model, followed by Tukey’s multiple-comparison test. For the ACS dose–response experiment, one-way ANOVA followed by Tukey’s multiple-comparison test was used. For time-dependent vacuole formation and Bafilomycin A1 dose–response analysis, homogeneity of variance was assessed using Levene’s test. As the assumption of equal variances was violated, Welch’s one-way ANOVA followed by Games–Howell post hoc multiple comparisons was performed. Statistical significance was defined as *p* < 0.05. Significance levels were denoted as * *p* < 0.05, ** *p* < 0.01, and *** *p* < 0.001; ns, *p* ≥ 0.05.

## 4. Conclusions

**Figure 12:**
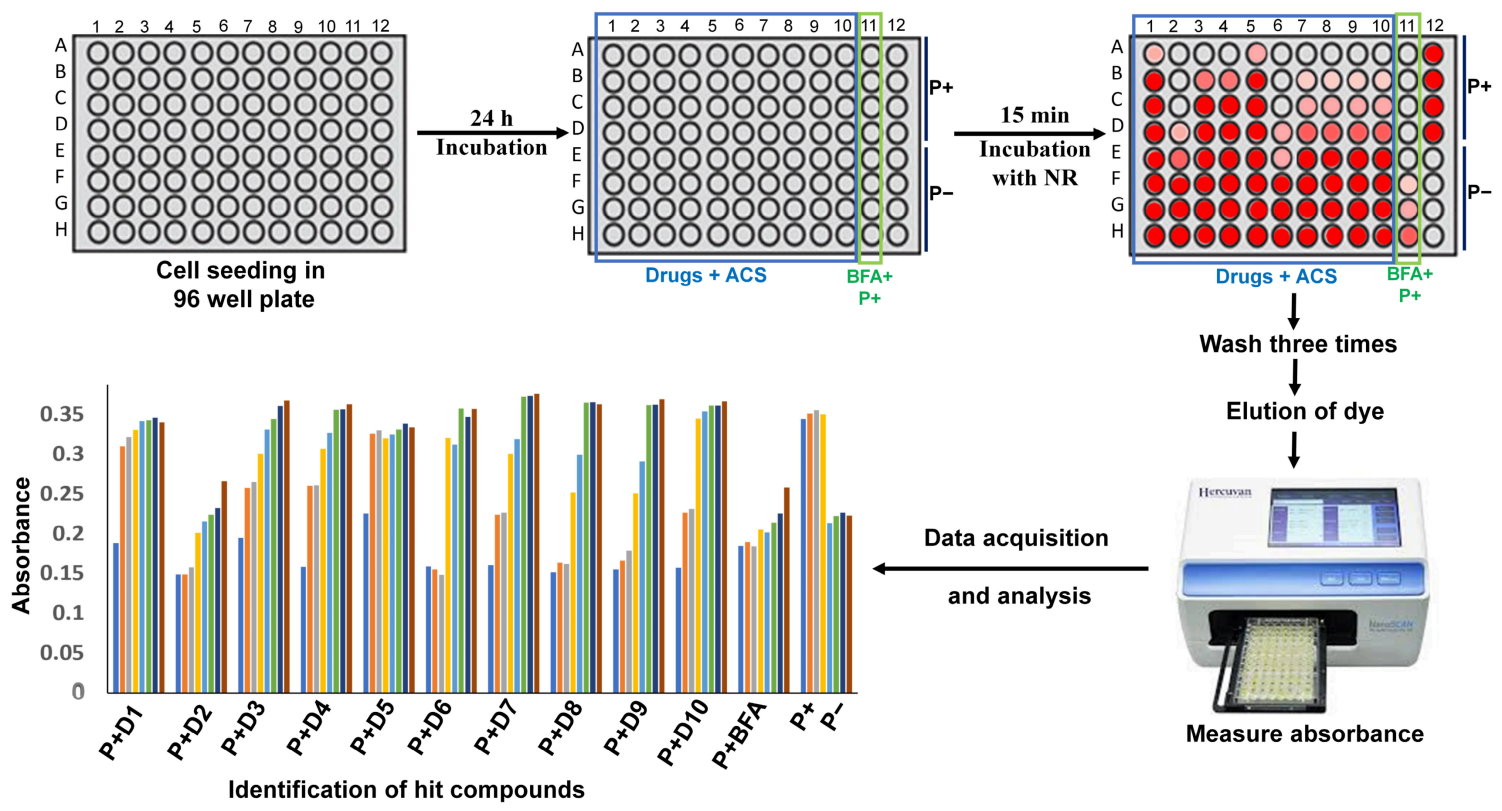
High-throughput drug screening based on vacuole quantification in ACS-treated cells. Schematic representation of the experimental workflow used for pharmacological screening of compounds that modulate activated calcium sulfate (ACS)–induced vacuolation. Cells were first seeded in a 96-well microplate and allowed to adhere under standard culture conditions. Cells were treated with different drugs (D1– D10) in lanes 1–10, followed by exposure to activated calcium sulfate (ACS) particles to induce vacuole formation. Lane 11 received Bafilomycin A1 (BFA1) prior to ACS treatment and served as the positive control for vacuole inhibition. Lane 12 served as the particle control, where the first four wells (A–D) received ACS particles (P+) while the remaining four wells (E–H) were left untreated (P−). Vacuole formation was subsequently quantified using a NR (neutral red) assay, and absorbance values were measured using a microplate reader. The resulting data were analyzed to compare the effects of different compounds on ACS-induced vacuolation.

Activated calcium sulfate (ACS) provides a biocompatible, chemically simple, and cost-effective alternative to conventional phagocytic particles and commercial assay kits for interrogating phagosome–lysosome dynamics. Unlike polymeric or opsonized particles that depend on receptor-specific uptake or immune priming, ACS induces robust vacuolization, enabling unbiased assessment of phagocytic pathways in professional and non-professional phagocytes. ACS-induced vacuoles undergo progressive maturation and acidification, permitting dynamic evaluation of lysosomal function using straightforward colorimetric readouts such as neutral red, without the requirement for specialized probes or instrumentation [54]. Building on these properties, the ACS-based drug screening assay described herein enables rapid, sensitive, and economical high-throughput identification of compounds that modulate ACS phagocytosis and vacuole formation, offering a practical advantage over imaging- and flow cytometry-dependent approaches (Figure 12). While the assay efficiently captures overall phagocytic activity, ACS represents an inorganic particle and does not fully recapitulate the biological complexity of phagocytic targets such as bacteria or yeast. To address this limitation, we are developing a second-generation hybrid particle system that integrates ACS with bacterial and yeast components, enabling comparative analysis of inorganic, bacterial, and fungal phagocytosis using the same simple neutral red and acridine orange (NR/AO)–based readout.

## 5. Patents

The corresponding author, Subrata K. Das, and author Alpana Joshi are inventors of a granted patent entitled *“Heat-Activated Calcium Sulfate Particles for Phagocytosis Assay and Method for Preparation Thereof”* (Indian Patent No. 583072), granted to Shobhit Institute of Engineering and Technology (Deemed-to-be University).

## Data Availability

Data will be made available on request.

## Supplementary Materials

The following supporting information can be downloaded at: https://www.mdpi.com/article/10.3390/molecules31214022/s1, Figure S1: Particle heterogeneity and vacuolation during time-resolved sedimentation; Table S1: List of drugs used for vacuole modulation screening in ACS-treated HeLa cells; Video S1: Time-lapse video of A549 cells treated with 2 min stock sedimentation heterogeneous ACS particles.

## Abbreviations

The following abbreviations are used in this manuscript:

ACS	Activated Calcium Sulfate
NR	Neutral Red
BFA1	Bafilomycin A1
V-ATPase	Vacuolar-type H^+^-ATPase
FT-IR	Fourier-Transform Infrared Spectroscopy
XRD	X-ray Diffraction
