# Supplementary material for: Activated CaSO4-Induced Vacuolation as a Quantitative Platform for Phagocytosis-Driven Drug Screening
Source: Molecules. 2026 Nov 8;31(21):4022. doi: 10.3390/molecules31214022 (PMC13765058; doi:10.3390/molecules31214022)
Supplement: Supplementary file 1 [file molecules-31-04022-s001.zip › molecules-4600059-Table S1.pdf]

Table S1. List of drugs used for vacuole modulation screening in ACS-treated HeLa cells.

Sl No	Drug (product)	Active ingredient(s)	Content per tablet/formulation (mg)	Pharmacological class	Concentration used in cell study (mg/200 µL)*
1	Ecosprin-75	Aspirin	75	NSAID (COX inhibitor); antiplatelet	0.53
2	Zerodol-SP	Aceclofenac + paracetamol + serratiopeptidas e	100 + 325 + 15	NSAID/analgesic with proteolytic enzyme	3.15
3	Galpride M1	Glimepiride + metformin	1 + 500	Sulfonylurea + biguanide (antidiabetic)	3.6
4	Zycel	Celecoxib	200	COX-2-selective NSAID	1.4
5	Atorlip	Atorvastatin	10	Statin (HMG-CoA reductase inhibitor)	0.07
6	Otrivin Oxy	Oxymetazoline	5	α-adrenergic agonist (nasal decongestant)	0.25
7	Pred Forte	Prednisolone acetate	50	Corticosteroid	0.5
8	Tropag-P	Phenylephrine + tropicamide	250 + 40	α1-adrenergic agonist + antimuscarinic (mydriatic)	2.9
9	O2 Tablets	Ofloxacin + ornidazole	200 + 500	Fluoroquinolone + nitroimidazole (antimicrobial)	5
10	BE-Fame-SP	Aceclofenac + paracetamol + serratiopeptidas e	100 + 325 + 15	NSAID/analgesic with proteolytic enzyme	3.15

A panel of 11 pharmacological agents with diverse therapeutic classes was selected for screening. The table details the drug name and the administered concentration, expressed in milligram. These compounds were screened for their ability to modulate vacuole formation and neutral red uptake in HeLa cells following treatment with activated calcium sulfate (ACS).
